# Suppression of tumor-associated neutrophils by lorlatinib attenuates pancreatic cancer growth and improves treatment with immune checkpoint blockade

**DOI:** 10.1038/s41467-021-23731-7

**Published:** 2021-06-07

**Authors:** Sebastian R. Nielsen, Jan E. Strøbech, Edward R. Horton, Rene Jackstadt, Anu Laitala, Marina C. Bravo, Giorgia Maltese, Adina R. D. Jensen, Raphael Reuten, Maria Rafaeva, Saadia A. Karim, Chang-Il Hwang, Luis Arnes, David A. Tuveson, Owen J. Sansom, Jennifer P. Morton, Janine T. Erler

**Affiliations:** 1 Biotech Research and Innovation Centre (BRIC), University of Copenhagen (UCPH), Copenhagen, Denmark; 2 CRUK Beatson Institute, Garscube Estate, Glasgow, UK; 3 Cold Spring Harbor Laboratory, Cold Spring Harbor, New York, NY USA; 4 Lustgarten Pancreatic Cancer Research Laboratory, Cold Spring Harbor, New York, NY USA; 5 Department of Microbiology and Molecular Genetics, University of California Davis, Davis, CA USA; 6 Institute of Cancer Sciences, University of Glasgow, Garscube Estate, Glasgow, UK

**Keywords:** Cancer microenvironment, Pancreatic cancer, Tumour immunology, Tumour immunology

## Abstract

Pancreatic ductal adenocarcinoma (PDAC) patients have a 5-year survival rate of only 8% largely due to late diagnosis and insufficient therapeutic options. Neutrophils are among the most abundant immune cell type within the PDAC tumor microenvironment (TME), and are associated with a poor clinical prognosis. However, despite recent advances in understanding neutrophil biology in cancer, therapies targeting tumor-associated neutrophils are lacking. Here, we demonstrate, using pre-clinical mouse models of PDAC, that lorlatinib attenuates PDAC progression by suppressing neutrophil development and mobilization, and by modulating tumor-promoting neutrophil functions within the TME. When combined, lorlatinib also improves the response to anti-PD-1 blockade resulting in more activated CD8 + T cells in PDAC tumors. In summary, this study identifies an effect of lorlatinib in modulating tumor-associated neutrophils, and demonstrates the potential of lorlatinib to treat PDAC.

## Abstract (web summary)

Tumor associated neutrophils have been correlated with poor prognosis in patients with pancreatic ductal adenocarcinoma (PDAC). Here the authors show that the tyrosine kinase inhibitor lorlatinib modulates neutrophil development and recruitment in the tumor microenvironment, attenuating PDAC progression in preclinical mouse models.

## Introduction

Pancreatic ductal adenocarcinoma (PDAC) has a dismal survival prognosis with only 8% of patients surviving for more than 5 years, largely due to late diagnosis and insufficient therapeutic options^1^. PDAC tumors and metastatic lesions are both characterized by excessive deposition of the extracellular matrix (ECM), and a tumor microenvironment (TME) containing fibroblasts and different immune cells^2–4^. Macrophages and neutrophils, the most abundant immune cells within the PDAC TME, are associated with a poor clinical prognosis due to their immunosuppressive properties and roles in mediating therapeutic resistance^5–8^. While several strategies that target macrophages are currently being evaluated in clinical trials^9,10^, few therapies that target the function of tumor-associated neutrophils are available^11^. In the metastatic setting, neutrophils (as well as other cell types) are responsible for the establishment of a hospitable niche that promotes the growth of disseminated PDAC cells^12,13^. In addition, a high neutrophil-to-lymphocyte ratio in patient blood samples is a prognostic marker for decreased postoperative survival in PDAC patients^14^. Neutrophils are produced in the bone marrow (BM) from hematopoietic progenitor cells and are recruited to tumors by tumor cell-derived cytokines and chemokines^15^, where they then promote tumor growth through the production of cytokines and secretion of ECM-modulating enzymes^15^. In addition, neutrophils support the survival of circulating tumor cells and promote the establishment of metastatic lesions by enhancing cancer stem cell features of metastasis-initiating cells and by suppressing CD8^+^ T-cell and NK cell cytotoxicity at the metastatic site^16–19^. However, neutrophils with anti-tumorigenic properties have also been described^20,21^, suggesting that the modulation of the recruitment and/or specific functions of tumor-associated neutrophils could be an effective therapeutic strategy for cancer patients.

Changes to the TME stimulate intracellular signaling pathways that enable cells to respond accordingly. One major mechanism of signaling is protein phosphorylation mediated by protein kinases. There are 518 protein kinases in the human body^22^ and many anticancer agents target kinase signaling^23^. Lorlatinib (PF-06463922) is an FDA-approved, third-generation, ATP-competitive small-molecule tyrosine kinase inhibitor that shows promising results in pre-clinical tests and clinical trials in patients suffering from non-small-cell lung cancer with genetic rearrangements of anaplastic lymphoma kinase (*ALK*) or *ROS1*^24,25^. Lorlatinib can also potently inhibit other tyrosine kinases, such as the non-receptor tyrosine kinase FES^26^.

Here, our goal was to uncover neutrophil targeting therapies through analysis of tumor-activated kinase signaling in neutrophils. We identify the non-receptor tyrosine kinase FES as a possible target to suppress neutrophils and prevent PDAC progression. In pre-clinical mouse models of PDAC, we discover that lorlatinib indirectly suppresses the growth of PDAC at primary and metastatic sites by modulating neutrophil development and recruitment from the BM and by suppressing neutrophil-induced tumor growth within the TME. Finally, we find that lorlatinib improves the response to anti-PD-1 blockade in PDAC tumors.

## Results

### PDAC cells activate FES kinase in neutrophils, which can be inhibited by lorlatinib

**Fig. 1:**
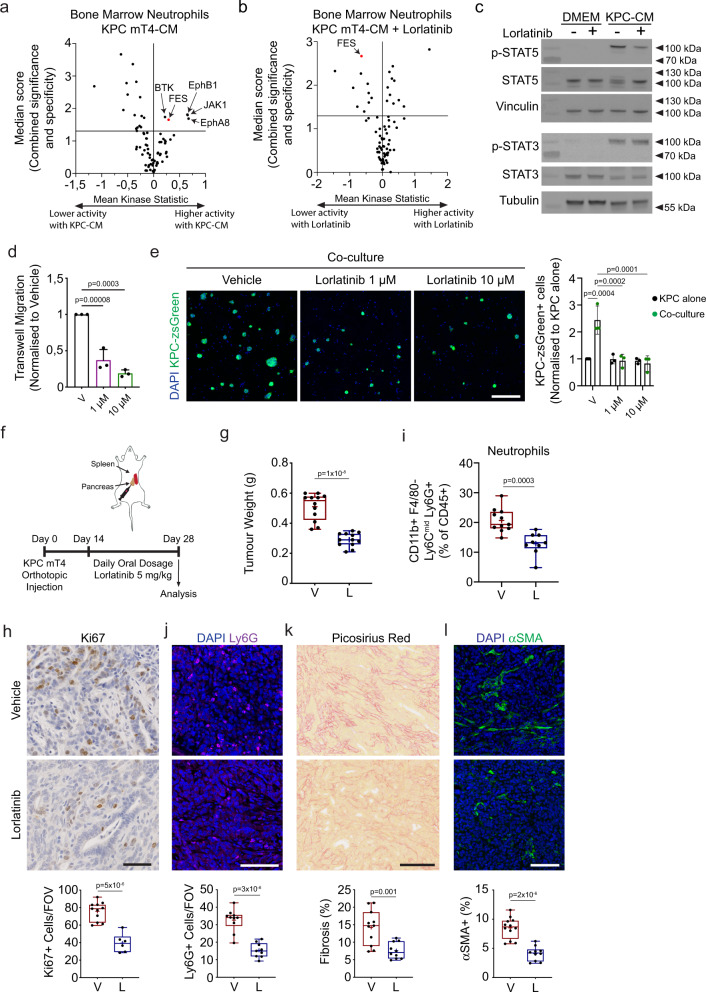
PDAC cells activate FES kinase signaling in neutrophils, which can be inhibited with lorlatinib to suppress neutrophil accumulation in PDAC tumors. **a**, **b** Volcano plots of the kinase activity prediction in neutrophils stimulated with (**a**) control or KPC-conditioned medium (KPC-CM) or (**b**) KPC-CM for 1 h in the presence of vehicle or 10 µM lorlatinib (data show results from three independent experiments). **c** Immunoblot analysis of p-STAT5, STAT5, p-STAT3, and STAT3 in neutrophils stimulated with control or KPC-CM in the presence of vehicle or lorlatinib (immunoblots are representative of one experiment performed three times). Sample integrity controls are vinculin and tubulin, respectively. Unprocessed scans of immunoblots are shown in Supplementary Fig. 2. **d** Transwell migration of neutrophils towards KPC-CM in presence of vehicle (V), 1 μM or 10 μM lorlatinib (*n* = 3 independent experiments). **e** Representative immunofluorescence images of neutrophil/KPC-zsGreen co-cultures in the presence of vehicle or lorlatinib after 2 days of culture (*n* = 3 independent experiments). **f** Orthotopic implantation of KPC mT4 cells into the pancreas. Mice were treated daily with vehicle (V) or 5 mg/kg lorlatinib (L) for 2 weeks starting 14 days after implantation. **g** Tumor weight of orthotopic PDAC tumors after treatment with vehicle or 5 mg/kg lorlatinib (*n* = 12 mice per condition; data are from two independent experiments). **h** Representative IHC images of Ki67 in tumors from mice treated with vehicle control or lorlatinib (*n* = 11 mice, vehicle; *n* = 6 mice, lorlatinib). Data are from two independent experiments. **i** Flow cytometry analysis of neutrophils in PDAC tumors from mice treated with vehicle or lorlatinib (*n* = 11 mice, vehicle; *n* = 9 mice, lorlatinib; data are from two experiments). **j**–**l** Representative images of (**j**) neutrophils (Ly6G^+^; purple) (*n* = 10 mice, vehicle; *n* = 9 mice, lorlatinib), (**k**) picrosirius red (*n* = 11 mice, vehicle; *n* = 10 mice, lorlatinib), and (**l**) fibroblasts (αSMA^+^; green) (*n* = 12 mice, vehicle; *n* = 10 mice, lorlatinib) in tumors from mice treated with vehicle control or lorlatinib. Data are from two independent experiments. Scale bars represent 100 µm (**h**, **j**–**l**) or 200 µm (**e**). Box-and-whisker plot shows the median (line), mean (plus sign), 25th and 75th percentiles (box), and 5th and 95th percentiles (whiskers); bar graphs represent mean and standard deviation; hypothesis testing performed using two-way ANOVA with Tukey’s method for multiple comparisons (**d**, **e**) or unpaired two-sided Student’s *t* test (**g**–**l**). Source data are provided as a Source Data file.

To determine potential signaling pathways in neutrophils that are activated in PDAC and might serve as therapeutic targets to modulate neutrophil function in cancer, we used a commercially available kinase profiling array to monitor the phosphorylation of 196 tyrosine bait peptides. We isolated the BM from healthy mice, enriched for neutrophils by magnetic depletion (Supplementary Fig. 1a), and then stimulated the cells with conditioned medium from the pancreatic cancer cell line KPC mT4 (KPC-CM) for 1 h and used the neutrophil protein lysate for kinase profiling analysis. Based on the peptide phosphorylation levels, the activity of corresponding kinases was predicted by a group-based prediction system^27,28^. We found that the non-receptor tyrosine kinases FES, BTK, JAK1, EphA8, and EphB1 were activated in neutrophils stimulated with KPC-CM (Fig. 1a). Inhibition of JAK or BTK signaling has previously been demonstrated in pre-clinical PDAC models^29–31^. We also isolated bone marrow neutrophils from the KPC mouse model (*Kras*^*LSL-G12D/+*^*;Trp53*^*LSL-R172H/+*^*;Pdx1–Cre*) with pancreatic tumors and demonstrated that FES was among the group of kinases with significant increased activity compared to neutrophils from tumor-free control mice (Supplementary Fig. 1b). Interestingly, it was recently reported that lorlatinib (PF-06463922), an FDA-approved inhibitor of ALK, potently inhibits FES^26^. To test if lorlatinib could inhibit FES activity in neutrophils, we stimulated neutrophils with KPC-CM in the presence of lorlatinib or vehicle for 1 h and used the profiling array to determine kinase activity. We found that lorlatinib reduced the activity of FES based on the phosphorylation pattern of the bait peptides in the array (Fig. 1b).

FES has been associated with signaling through STAT proteins^32^, and both STAT3 and STAT5 play a role in neutrophil development and homeostasis^33–35^. Therefore, we hypothesized that lorlatinib inhibition of FES signaling would result in reduced phosphorylation of STAT proteins. Indeed, we observed reduced phosphorylation of STAT5 when we added lorlatinib to neutrophils stimulated with KPC-CM, whereas no change was observed in the phosphorylation of STAT3 (Fig. 1c, S2a–e). We determined that *Fes* is expressed in CD11b + -sorted myeloid cells from an orthotopic PDAC tumor (Supplementary Fig. 1c) and we further confirmed that *Fes* is expressed at the highest level in neutrophils by analyzing *Fes* expression in neutrophils (Ly6G^+^), monocytes (Ly6C^+^) and macrophages (F4/80^+^) sorted from pancreatic tumors from the KPC mouse model (Supplementary Fig. 1d). To determine how lorlatinib modulates neutrophils we tested neutrophil migration by seeding freshly isolated BM neutrophils in transwell inserts with KPC-CM as a chemoattractant in the bottom chamber. We observed that lorlatinib reduces neutrophil migration in response to KPC-CM compared to vehicle (Fig. 1d). To explore the influence of lorlatinib-treated neutrophils on cancer cell growth, we cultured zsGreen-expressing KPC mT4 cells together with freshly isolated BM neutrophils in KPC-CM in the presence of lorlatinib or vehicle. After 2 days of co-culture, we fixed the cells and quantified the number of zsGreen^+^ cells. While we did not see any effect of lorlatinib on the number of zsGreen+ cells without neutrophils present, we found that lorlatinib reduced the number of KPC mT4 cells in neutrophil co-cultures compared to vehicle (Fig. 1e, S1e). We confirmed that lorlatinib does not inhibit the proliferation of KPC cells using a cell viability assay (Supplementary Fig. 1f). Finally, to confirm that the effect of lorlatinib on neutrophil function is not caused by directly inducing cell death, we isolated BM neutrophils and determined their viability in the presence of lorlatinib after 2 days of cell culture. As reported previously^36^, neutrophil viability declines very fast in regular cell culture medium and was not detectable at day 2. However, we were able to rescue neutrophil viability when cells were cultured in either KPC-CM, GM-CSF or G-CSF (Supplementary Fig. 1g). Importantly, the presence of lorlatinib in either condition did not reduce neutrophil viability (Supplementary Fig. 1g). Taken together, these results suggest that PDAC cells induce FES activity in neutrophils in vitro, and that lorlatinib could be used to target tumor-associated neutrophils in PDAC.

### Lorlatinib attenuates PDAC growth by suppressing neutrophil accumulation

Lorlatinib shows promising results in clinical trials with patients suffering from non-small-cell lung cancer with genetic rearrangement of *ALK* or *ROS1*^24,25^, however, only a very small percentage of human PDAC patients have detectable ALK expression^37^. Based on our results on neutrophils in vitro, we decided to test whether lorlatinib could inhibit PDAC tumor growth in vivo. We injected KPC mT4 cells into the pancreas of healthy syngeneic mice and started the treatment of established tumors after 14 days with a daily oral dose of lorlatinib (5 mg/kg) for a further 14 days. Mice were euthanized 28 days after tumor cell injection and analyzed (Fig. 1f). The weight of PDAC tumors was significantly reduced in lorlatinib-treated mice compared to vehicle-treated mice (Fig. 1g, S3a). When tumors were examined by IHC staining for markers of proliferation (Ki67) and apoptosis (cleaved caspase 3), we observed a reduction in proliferation (Fig. 1h), but no difference in apoptosis (Supplementary Fig. 3b). PDAC tumors are characterized by excessive deposition of ECM, which is believed to affect treatment efficacy by limiting drug delivery, and a high degree of immune cell infiltration^3^. To determine whether lorlatinib modulates these aspects of the PDAC TME, we performed flow cytometry (Supplementary Fig. 3c–e) and IHC staining to evaluate immune cell composition and fibrosis, respectively, of tumors from vehicle- or lorlatinib-treated mice. We did not detect any difference in the percentage of F4/80^+^ macrophages, Ly6C^+^ monocytes, Nk1.1^+^ NK cells or CD3^+^ T cells among CD45^+^ immune cells in the tumor (Supplementary Fig. 3f). In contrast, we detected a significant increase in B220^+^ B cells and a reduction in Ly6G^+^ neutrophils in lorlatinib-treated mice (Fig. 1i, Supplementary Fig. 3f). Since B cells are mainly pro-tumorigenic in PDAC^38^, we focused on the effect of lorlatinib on neutrophils. We confirmed by immunofluorescence staining of tumor sections that lorlatinib treatment reduced the number of neutrophils in PDAC tumors (Fig. 1j). Next, we analyzed the tumors for fibrosis by IHC. We found that lorlatinib-treated mice had markedly reduced levels of fibrosis, as seen by both decreased collagen deposition and reduced numbers of αSΜΑ^+^ fibroblasts compared to vehicle-treated mice (Fig. 1k,l). Combined with our results obtained with neutrophils in vitro, these data suggest that lorlatinib inhibits PDAC progression by suppressing neutrophil accumulation and reducing tumor-associated fibrosis in the TME.

### Lorlatinib reduces metastatic growth in the liver

**Fig. 2:**
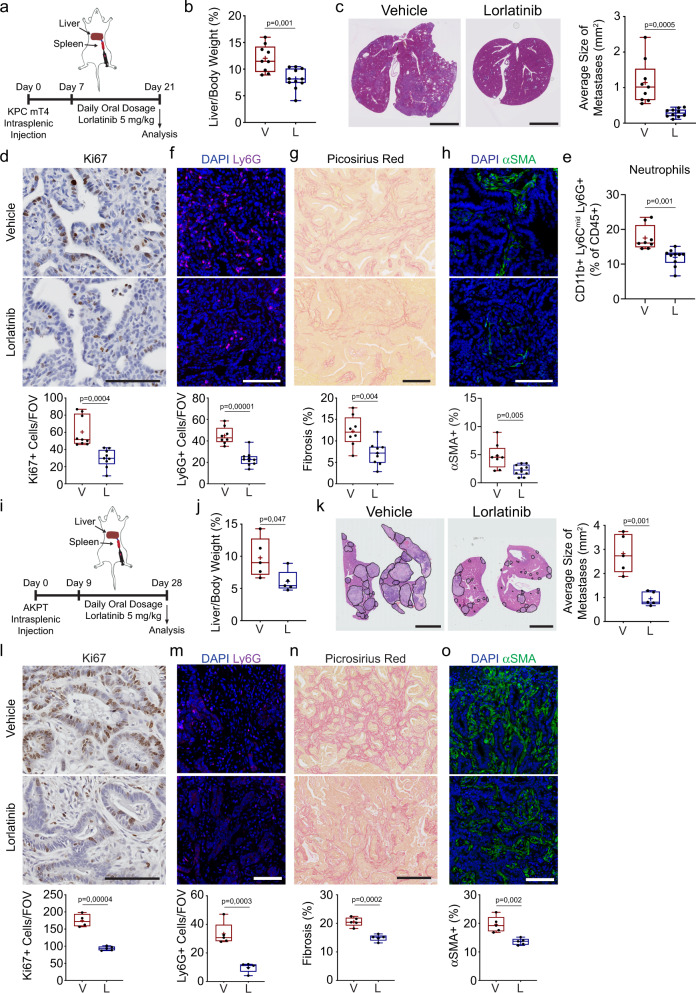
Lorlatinib treatment attenuates progression of hepatic PDAC and CRC metastases. **a** Intrasplenic injection of KPC mT4. Mice were treated daily with vehicle (V) or 5 mg/kg lorlatinib (L) for 2 weeks starting 7 days after implantation. **b**, **c** Quantification of hepatic metastatic burden after vehicle or lorlatinib treatment by (**b**) liver weight (%) of total body weight and (**c**) hematoxylin & eosin staining and average size of metastatic lesions (*n* = 9 mice, vehicle; *n* = 11 mice; data are from two independent experiments). **d** Representative IHC images of Ki67 (*n* = 9 mice per group). Data are from two independent experiments. **e** Flow cytometry analysis of neutrophils (CD11b^+^ F4/80^neg^ Ly6C^neg^ Ly6G^+^) in hepatic metastases from vehicle- or lorlatinib-treated mice (*n* = 8 mice, vehicle; *n* = 10 mice, lorlatinib; data are from two independent experiments). **f**–**h** Representative images of (**f**) neutrophils (Ly6G^+^; purple) (*n* = 8 mice, vehicle; *n* = 10 mice, lorlatinib), (**g**) picrosirius red (*n* = 8 mice, vehicle; *n* = 9 mice, lorlatinib), and (**h**) fibroblasts (αSMA^+^; green) (*n* = 8 mice, vehicle; *n* = 10 mice, lorlatinib) in hepatic metastases from vehicle- and lorlatinib-treated mice. Data are from two independent experiments. **i** Intrasplenic injection of CRC organoids from *villin*CreER *Apc*^*fl/fl*^ *Kras*^*G12D/+*^ *Trp53*^*fl/R172H*^ *TgfbrI*^*fl/fl*^ (AKPT). Mice were treated with vehicle or 5 mg/kg lorlatinib starting 9 days after injection. Hepatic metastatic burden was determined 20 days after the start of treatment. **j**, **k** Quantification of hepatic metastases in mice after intrasplenic injection of CRC organoids and vehicle or lorlatinib treatment by (**j**) liver weight (%) of total body weight and (**k**) representative hematoxylin & eosin staining with quantification of the average metastatic lesion size (*n* = 5 per group; data are from one experiment). Black lines show the outline of individual metastatic lesions. **l**–**o** Representative IHC and immunofluorescence images of (**l**) Ki67 (*n* = 4 mice, vehicle; *n* = 5 mice, lorlatinib), (**m**) neutrophils (Ly6G^+^; green) (*n* = 5 mice per group), (**n**) picrosirius red (*n* = 5 mice per group), and (**o**) fibroblasts (αSMA^+^; green) (*n* = 5 mice per group) in hepatic metastases from vehicle- and lorlatinib-treated mice. Data are from one experiment. Scale bars represent 5 mm (**c**, **k**) or 100 µm (**d**, **f**–**h**, **l**–**o**). Box-and-whisker plot shows the median (line), mean (plus sign), 25th and 75th percentiles (box), and 5th and 95th percentiles (whiskers); hypothesis testing performed using unpaired two-sided Student’s *t* test (**b**–**o**). Source data are provided as a Source Data file.

Currently, the best treatment option for PDAC patients is the surgical removal of the pancreatic tumor. Unfortunately, by the time PDAC patients are diagnosed, most (∼80%) present with non-resectable tumors and/or metastatic disease. Moreover, surgically-resected patients that relapse with distant hepatic recurrence have a significantly reduced median recurrence-free survival compared to patients with all other recurrence patterns (i.e., local or lung)^39,40^. To assess if lorlatinib could suppress the growth of PDAC metastatic lesions in the liver, we used an experimental liver metastasis model. We injected KPC mT4 cells into the spleen of healthy syngeneic mice. In this model, tumor cells drain directly from the spleen into the liver through the portal circulation and generate metastases restricted to the liver^12^. We started treatment with lorlatinib 7 days after the intrasplenic injection and continued daily treatment for 14 days, after which metastases were analyzed (Fig. 2a). We found that the liver weight relative to the body weight was reduced in mice treated with lorlatinib compared to vehicle-treated mice (Fig. 2b). We further quantified the size and number of metastatic lesions in the liver and found a significant decrease in size but not the number of metastatic lesions in livers of lorlatinib-treated mice (Fig. 2c and S4a), which leads to a significant reduction in metastatic burden in lorlatinib-treated mice (Supplementary Fig. 4b). Similar to our results from the orthotopic model, we evaluated proliferation and apoptosis by IHC and observed a reduction in proliferative cells in liver metastases from lorlatinib-treated mice (Fig. 2d), but no difference in apoptosis (Supplementary Fig. 4c), compared to vehicle-treated mice. These results suggest, similar to the orthotopic model, that lorlatinib suppresses the growth of metastatic lesions in the liver. To assess the composition of immune cells in lorlatinib-treated metastases, we prepared single-cell suspensions and performed flow cytometry analysis. We did not detect any difference in the percentage of F4/80^+^ macrophages, Ly6C^+^ monocytes, B220^+^ B cells, Nk1.1^+^ NK cells, CD4^+^ or CD8^+^ T cells among CD45^+^ immune cells in the tumor (Supplementary Fig. 4d). In contrast, we detected a significant reduction in Ly6G^+^ neutrophils in lorlatinib-treated mice (Fig. 2e). We confirmed by immunofluorescence staining of tumor sections that lorlatinib treatment reduced the number of neutrophils in hepatic metastases (Fig. 2f). Metastatic lesions from pancreatic cancer are characterized by excessive deposition of ECM similar to the primary tumor site^12^. Similarly, we found that metastatic lesions in lorlatinib-treated mice had markedly reduced levels of fibrosis, as seen by both decreased collagen deposition and reduced numbers of αSMA^+^ cells compared with metastatic lesions from vehicle-treated mice (Fig. 2g, h). To confirm that reduced neutrophil influx, fibrosis, and fibroblast accumulation are not simply due to the presence of smaller metastases in lorlatinib-treated mice, we analyzed size-matched lesions by IHC staining. We found that both neutrophil and fibroblast accumulation in size-matched metastases was reduced in mice treated with lorlatinib, whereas the reduction in fibrosis was not significant (Supplementary Fig. 4e). Taken together, these results suggest that lorlatinib inhibits PDAC tumor growth both at the primary tumor site and at a secondary site, by suppressing tumor-associated neutrophils and cancer-associated fibroblasts (CAFs), the latter possibly as an indirect effect of the inhibition of neutrophils.

While we cannot exclude a direct effect on tumor cells, our results suggest that the main effect of lorlatinib is on cells of the TME. Therefore, we hypothesized that lorlatinib treatment would be applicable in other cancer types with a heterogenous TME composition. Given that the liver is a common site of metastasis for other gastrointestinal cancers such as colorectal cancer (CRC), we determined if lorlatinib treatment could also inhibit metastatic progression by CRC cells. To test this, we prepared single-cell suspensions from small colorectal tumor-derived organoids from *villin*CreER *Apc*^*fl/fl*^*Kras*^*G12D/+*^*Trp53*^*fl/R172H*^*TgfbrI*^*fl/fl*^ (AKPT) mice^41^ and injected them directly into the spleen of healthy syngeneic mice. We started lorlatinib treatment 9 days after the intrasplenic injections and administered daily treatment for 20 days, after which, we analyzed metastatic burden by IHC (Fig. 2i). We found that the liver weight relative to the body weight was reduced in mice treated with lorlatinib compared to vehicle-treated mice (Fig. 2j). We further quantified the number and size of metastatic lesions in the liver, and found a significant decrease in size of metastatic lesions in livers of lorlatinib-treated mice, while there was no significant difference in the number of metastatic lesions (Fig. 2k and S4f). We also evaluated proliferation and apoptosis by IHC, and observed similar results as in our previous models (Fig. 2l and S4g). Taken together, these results suggest that lorlatinib affects the growth of metastatic CRC lesions. Similar to the results from our PDAC metastasis model, we found that the CRC metastatic lesions in lorlatinib-treated mice had a reduced number of neutrophils (Fig. 2m). Finally, we found reduced levels of fibrosis in CRC metastatic lesions from lorlatinib-treated mice, as seen by both decreased collagen deposition and reduced numbers of αSMA^+^ fibroblasts, compared with metastatic lesions from vehicle-treated mice (Fig. 2n, o). Taken together, these results suggest that lorlatinib inhibits tumor growth both at the primary site and a secondary site, in different tumor types by modulating neutrophils and the TME.

### Lorlatinib targets neutrophils to limit PDAC progression

**Fig. 3:**
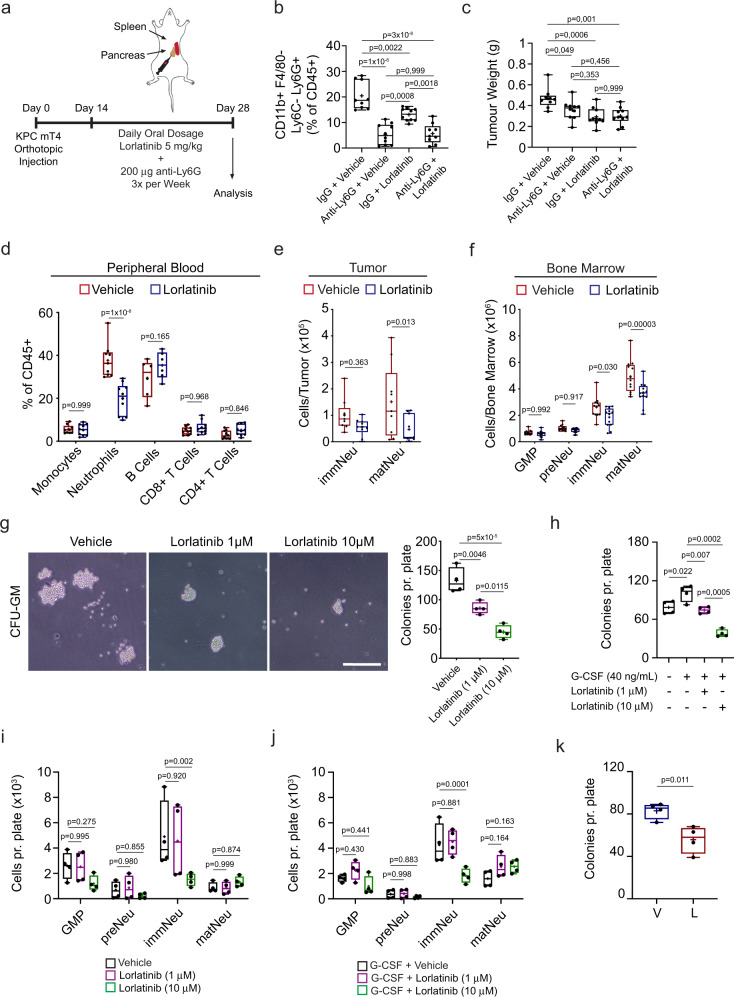
Lorlatinib treatment suppresses neutrophil development. **a** Orthotopic implantation of KPC mT4 cells into the pancreas. Mice were treated daily with combinations of vehicle (V), 5 mg/kg lorlatinib (L), 200 μg control IgG and anti-Ly6G. **b** Flow cytometry analysis of neutrophils in PDAC tumors from mice treated with a combination of vehicle, lorlatinib, control IgG and anti-Ly6G (*n* = 9 mice, vehicle + control IgG, and anti-Ly6G + lorlatinib groups; *n* = 10 mice, remaining groups). **c** Tumor weight of orthotopic PDAC tumors from mice treated with a combination of vehicle, lorlatinib, control IgG, and anti-Ly6G (*n* = 9 mice, vehicle + control IgG; *n* = 10 mice, remaining conditions). Data are from two experiments. **d** Flow cytometry analysis of immune cells at day 28 in blood samples from mice with PDAC tumors treated with vehicle or 5 mg/kg lorlatinib for 14 days (see outline of the experiment in Fig.1f) (*n* = 9 mice, vehicle; *n* = 10 mice, lorlatinib (monocytes/neutrophils); *n* = 10 mice per group (T cells); *n* = 6 mice per group (B cells); data are from two independent experiments). **e** Flow cytometry analysis of immature (immNeu) and mature (matNeu) neutrophils in PDAC tumors at day 28 after treatment with vehicle or lorlatinib for 14 days (*n* = 9 mice, vehicle; *n* = 10 mice, lorlatinib; data are from two independent experiments). **f** Flow cytometry analysis at day 28 of GMPs, pre-neutrophils (preNeu), immature and mature neutrophils in the bone marrow (BM) from mice with PDAC tumors treated with vehicle or lorlatinib for 14 days (*n* = 12 mice per group; data are from two independent experiments). **g** Colony-forming unit*–*granulocyte macrophage (CFU–GM) assay of BM cells from healthy mice with vehicle or lorlatinib. Colonies with >20 cells were quantified after 3 days (*n* = 4 independent experiments). **h**–**j** The number of colonies from CFU–GM (**h**) and flow cytometry analysis (**g**) of neutrophils on day 3 after CFU–GM culture in the (**i**) absence or (**j**) presence of recombinant G-CSF and lorlatinib (*n* = 4 independent experiments). **k** CFU–GM of BM cells isolated on day 28 from mice with PDAC tumors after daily treatment with vehicle or lorlatinib for 14 days (*n* = 4 mice per group). Scale bars represent 200 µm. Box-and-whisker plot shows the median (line), mean (plus sign), 25th and 75th percentiles (box), and 5th and 95th percentiles (whiskers); hypothesis testing performed using two-way ANOVA with Tukey’s (**b**, **c**, **g**, **h**) or Sidak’s (**d**–**f**, **i**, **j**) method for multiple comparisons or unpaired two-sided Student’s *t* test (**k**). Source data are provided as a Source Data file.

We sought to determine if neutrophils support tumor progression in our orthotopic PDAC model and if lorlatinib targets neutrophils by comparing neutrophil depletion with lorlatinib treatment. We implanted KPC mT4 cells into the pancreas of syngeneic mice and depleted neutrophils by administering an anti-Ly6G antibody every 3 days over 2 weeks, after which tumors were harvested for analysis. In addition, we combined neutrophil depletion with daily lorlatinib treatment to determine the effect of depleting neutrophils on the efficacy of lorlatinib treatment (Fig. 3a). As expected, neutrophils were significantly reduced in tumors of anti-Ly6G-treated animals as determined by flow cytometry (Fig. 3b) and IHC analysis (Supplementary Fig. 5a). Importantly, the weight of orthotopic PDAC tumors was significantly reduced after neutrophil depletion or lorlatinib treatment compared to mice treated with control IgG and vehicle (Fig. 3c). Interestingly, there was no increased benefit of depleting neutrophils in combination with lorlatinib treatment compared to either single treatment (Fig. 3c). Furthermore, we found that neutrophil depletion reduces proliferation as measured by the decrease of Ki67^+^ cells in tumors from neutrophil-depleted mice, but we did not observe any difference in Ki67^+^ cells when comparing neutrophil depletion with lorlatinib alone or the combination of neutrophil depletion and lorlatinib (Supplementary Fig. 5b). Finally, collagen deposition and the accumulation of αSΜΑ^+^ fibroblasts was also reduced in neutrophil-depleted mice through anti-Ly6G compared to control IgG and vehicle resulting in reduced tumor-associated fibrosis. Moreover, we did not observe further reductions in fibrosis when compared to treatment with lorlatinib alone or combined neutrophil depletion with lorlatinib (Supplementary Fig. 5c, d). To assess whether lorlatinib has a direct inhibitory effect on CAFs, we analyzed two independent CAF cell lines isolated from PDAC tumors in the KPC mouse model^42^, and found no effect of lorlatinib on either CAF proliferation, matrix contraction of collagen I gels with CAFs embedded, or gene expression of CAF activation markers, *Acta2* and *Col1a1*, when CAFs were stimulated with KPC-CM in the presence of lorlatinib (Supplementary Fig. 6a–c). These results suggest that lorlatinib specifically targets neutrophils to suppress PDAC since we do not see an added effect with lorlatinib in neutrophil-depleted mice.

### Lorlatinib suppresses neutrophil expansion in the BM

Neutrophils are produced daily in vast numbers through progressively differentiated precursors within the BM and are released into circulation^43^. A fundamental characteristic of this process is their release from the BM into the blood circulation and their migration to sites of inflammation. In cancer, tumor-secreted cytokines stimulate the development and release of neutrophils with an immunosuppressive and tumor-promoting phenotype into the circulation^44,45^. To gain a better understanding of how the pancreatic cancer cells modulate neutrophil function, we collected KPC-CM and analyzed cytokine and chemokine secretion. We found that KPC cells secrete several cytokines and chemokines, including CXCL1, G-CSF, and GM-CSF (Supplementary Fig. 7a), which are known neutrophil regulators^15^. To determine whether lorlatinib could modulate the response of neutrophils to these secreted factors, we investigated the ability of neutrophils to migrate towards each factor in the presence or absence of lorlatinib. We found that CXCL1, G-CSF, and GM-CSF induced neutrophil migration, however, this effect was abrogated for G-CSF and GM-CSF in the presence of lorlatinib, whereas lorlatinib did not affect neutrophil migration in response to CXCL1 (Supplementary Fig. 7b). To determine if reduced neutrophil accumulation in tumors is caused by a systemic effect of lorlatinib treatment and not just suppression of neutrophil migration into the tumor, we analyzed peripheral blood from tumor-bearing vehicle- or lorlatinib-treated mice and found a significant decrease of neutrophils in blood samples from lorlatinib-treated tumor-bearing mice (Fig. 3d). Development of neutrophils was recently described to progress from a proliferative neutrophil precursor population, termed pre-neutrophils, that gives rise to an intermediate population of immature neutrophils in the BM before differentiating into mature neutrophils^46^. Pre-neutrophils are cKit^+^, whereas immature neutrophils are cKit^−^Ly6G^+^CXCR2^−^ and mature neutrophils are cKit^−^Ly6G^+^CXCR2^+^. Based on our results, we hypothesized that lorlatinib affects neutrophil development in the BM. To evaluate this, we implanted KPC mT4 cells into the pancreas of healthy mice and started treatment with lorlatinib after 14 days for a further 2 weeks. We isolated and prepared single-cell suspensions from the BM and tumors from lorlatinib-treated mice and analyzed the maturation of neutrophils by flow cytometry using the markers described above (Supplementary Fig. 8a, b). We found that lorlatinib reduced the number of mature neutrophils in orthotopic PDAC tumors, while no significant difference was detected for immature neutrophils in the tumors (Fig. 3e). Interestingly, the numbers of both immature and mature neutrophils were reduced in the BM, while numbers of granulocyte–macrophage progenitors (GMP) and pre-neutrophils were not changed (Fig. 3f). We noted a higher expression of *FES* in neutrophil subsets in both mouse^46^ and human^47^ BM (Supplementary Fig. 8c, d). To determine if lorlatinib treatment reduces granulopoiesis, we evaluated the ability of freshly isolated BM cells from healthy mice to establish colonies in semi-solid methylcellulose medium supplemented with recombinant cytokines (SCF, IL-3, and IL-6). We counted established colonies 3 days after BM isolation and observed that lorlatinib reduced the number of colonies in a dose-dependent manner (Fig. 3g). Based on our results that lorlatinib does not affect the viability of neutrophils isolated from the BM (Supplementary Fig. 1g), we decided to test if lorlatinib instead inhibits the development of neutrophils. We isolated BM from healthy mice and seeded the cells in semi-solid methylcellulose with vehicle or lorlatinib as before. We included either G-CSF or GM-CSF to promote neutrophil development and cultured the cells for 3 days before we analyzed them by flow cytometry. We found an increased number of colonies after the addition of G-CSF and GM-CSF, but while lorlatinib was able to reduce colony formation after the addition of G-CSF at both 1 and 10 μM, it was only able to reduce colony formation after addition of GM-CSF at 10 μM (Fig. 3h and S8e). We observed no difference in GMPs, pre-neutrophils, or mature neutrophils after treatment with lorlatinib in control or after G-CSF stimulation, while lorlatinib decreased the number of immature neutrophils at 10 μM in control and G-CSF cultures (Fig. 3i, j). The decrease in immature neutrophils was not significant for GM-CSF-treated cultures (Supplementary Fig. 8f). Of note, we did not observe any difference in GMP or neutrophil subsets in the blood or BM from tumor-free mice treated with lorlatinib for 14 days (Supplementary Fig. 8g, h). These results suggest that lorlatinib suppresses tumor-induced granulopoiesis in the BM of tumor-bearing mice. Finally, to confirm that lorlatinib suppresses the tumor-induced expansion of neutrophils in the BM, we isolated the BM from vehicle- or lorlatinib-treated tumor-bearing mice and seeded the cells in a semi-solid methylcellulose medium. We detected significantly fewer colonies in the plates with BM cells from tumor-bearing lorlatinib-treated mice compared to cells from vehicle-treated mice (Fig. 3k and S8i). Taken together, these results suggest that lorlatinib suppresses the growth of PDAC at both the primary tumor and at metastatic sites by affecting neutrophils in three ways: (1) by modulating the development of neutrophils in the BM, (2) reducing their accumulation in the TME, and (3) by suppressing their ability to stimulate growth of PDAC cells in the TME.

### Lorlatinib extends survival of KPC mice

**Fig. 4:**
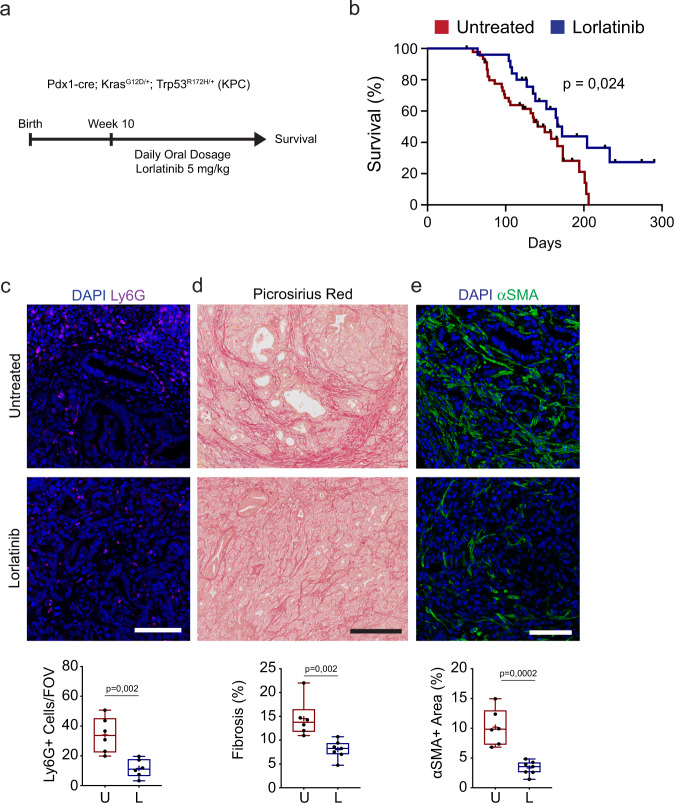
Lorlatinib treatment extends the survival of KPC mice. **a** KPC mice with spontaneous development of PDAC were treated daily with 5 mg/kg lorlatinib starting when mice reached 10 weeks of age until they developed signs of disease progression. **b** Kaplan–Meier survival plot of KPC mice treated from 10 weeks of age with lorlatinib (*n* = 25 mice) or untreated (*n* = 46 mice). Mice euthanized due to other pathologies are censored and cross-marked; *P* value by log-rank test. **c** Representative immunofluorescence images and quantification of neutrophils (Ly6G^+^; purple) in PDAC tumors collected from untreated or lorlatinib-treated KPC mice at the end of the experiment (*n* = 6 mice, untreated; *n* = 6 mice, lorlatinib; data are from one experiment). Five FOV were acquired per mouse and used for quantification of Ly6G^+^ cells. **d** Representative picrosirius red staining and quantification of fibrosis in PDAC tumors collected from untreated or lorlatinib-treated KPC mice at the end of the experiment (*n* = 6 mice, untreated; *n* = 7 mice, lorlatinib; data are from one experiment). **e** Representative immunofluorescence images and quantification of fibroblasts (αSMA^+^; green) in PDAC tumors collected from untreated or lorlatinib-treated KPC mice at the end of the experiment (*n* = 6 mice, untreated; *n* = 7 mice, lorlatinib; data are from one experiment). Scale bars represent 250 µm (**d**) or 100 µm (**c**, **e**). Box-and-whisker plot shows the median (line), mean (plus sign), 25th and 75th percentiles (box), and 5th and 95th percentiles (whiskers); hypothesis testing performed using unpaired two-sided Student’s *t* test. Source data are provided as a Source Data file.

To test the efficacy of lorlatinib in a spontaneous model of PDAC that recapitulates full disease progression and the histopathological features of human disease, we used the KPC mouse model. These mice have pancreas-specific mutations in *Kras*^*G12D*^ and *Trp53*^*R172H*^, and develop invasive, metastatic tumors that exhibit an extensive stroma with significant collagen deposition and infiltrating immune cells^48^. KPC mice were treated with lorlatinib (5 mg/kg) from 10 weeks of age (Fig. 4a). At this time, mice have widespread advanced pancreatic neoplasia^48^ and are more likely to mimic surgically resectable disease. Mice were monitored for signs of disease and euthanized when they became symptomatic of disease progression. We found that treatment with lorlatinib alone prolonged the survival of KPC mice with a median survival time of 172 days, compared to 150 days for control KPC mice (Fig. 4b). Similar to our orthotopic and intrasplenic models, we found that the primary tumors collected at end point (when mice show clear symptoms of PDAC) from lorlatinib-treated KPC mice had markedly reduced accumulation of Ly6G^+^ neutrophils by immunofluorescence staining (Fig. 4c). We also confirmed that lorlatinib treatment had markedly reduced levels of fibrosis, as seen by both decreased collagen deposition and reduced numbers of αSMA^+^ cells compared with untreated mice (Fig. 4d, e). While we observed reduced metastatic burden in our liver metastasis model, we did not observe any difference in metastatic burden in lorlatinib-treated KPC mice. However, our results demonstrate that lorlatinib treatment extends the survival of KPC mice, which is associated with modulation of the pancreatic TME, similarly to what we observed in the tumor transplantation models.

### Lorlatinib improves anti-PD-1 therapy

**Fig. 5:**
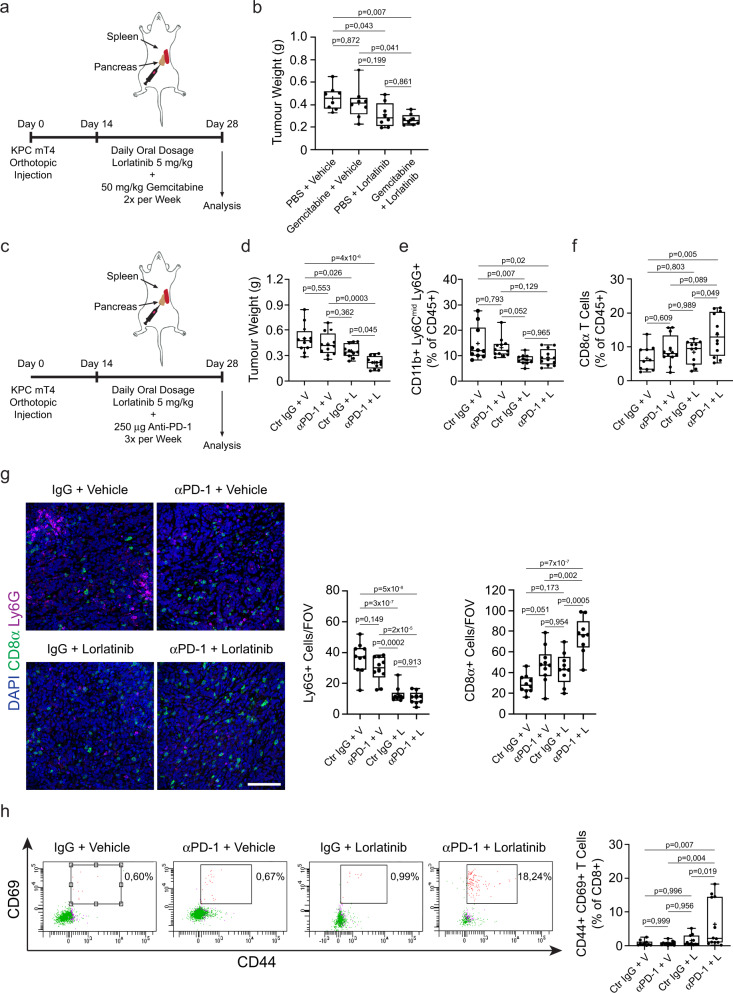
Lorlatinib treatment improves the response of PDAC tumors to immunotherapy, but not chemotherapy. **a** Orthotopic implantation of KPC mT4 cells into the pancreas. Mice were treated daily with vehicle (V) or 5 mg/kg lorlatinib (L) for 2 weeks and PBS or 50 mg/kg gemcitabine twice per week starting 14 days after implantation. **b** Weight of PDAC tumors from mice treated with vehicle or lorlatinib in combination with PBS or Gemcitabine (*n* = 8 mice per condition; data are from two independent experiments). **c** Orthotopic implantation of KPC mT4 cells into the pancreas. Mice were treated daily with vehicle (V) or 5 mg/kg lorlatinib (L) for 2 weeks and 250 μg control IgG or anti-PD-1 antibodies three times per week starting 14 days after implantation. **d** Weight of PDAC tumors from mice treated with combinations of vehicle, lorlatinib, control IgG, or anti-PD-1 (*n* = 12 mice per condition, except *n* = 11 mice for control IgG + lorlatinib condition; data are from three independent experiments). **e**, **f** Flow cytometry analysis of (**e**) neutrophils and (**f**) CD8^+^ T cells in PDAC tumors from mice treated with combinations of anti-PD-1 and lorlatinib (*n* = 10 mice, control IgG + vehicle; *n* = 11 mice, control IgG + lorlatinib; *n* = 12 mice, remaining groups; data are from three independent experiments). **g** Immunofluorescence analysis and quantification of neutrophils (Ly6G^+^; purple) and T cells (CD8^+^; green) cells in PDAC tumors treated for 14 days with combinations of anti-PD-1 and lorlatinib (*n* = 10 mice, control IgG + vehicle and anti-PD-1 + vehicle groups, *n* = 9 mice for remaining groups; data are from two independent experiments). **h** Flow cytometry plots and analysis of CD44^+^ CD69^+^ CD8^+^ T cells in tumors from mice treated with combinations of anti-PD-1 and lorlatinib (*n* = 10 mice, control IgG + vehicle; *n* = 11 mice, control IgG + lorlatinib; *n* = 12 mice, remaining groups; data are from three independent experiments). Scale bar represent 100 µm. Box-and-whisker plot shows the median (line), mean (plus sign), 25th and 75th percentiles (box), and 5th and 95th percentiles (whiskers); hypothesis testing performed using two-way ANOVA with Tukey’s method for multiple comparisons. Source data are provided as a Source Data file.

Recent studies have shown that modulation of TME components in PDAC can improve the therapeutic response to chemotherapy or immunotherapy^6,49^. To determine if lorlatinib could improve the efficacy of chemotherapy, we treated mice bearing orthotopic KPC tumors with gemcitabine (50 mg/kg) alone or in combination with lorlatinib (5 mg/kg) (Fig. 5a). Consistent with reported results^50^, we found that treatment with gemcitabine had little effect on the weight of orthotopic tumors. In contrast, similar to our previous results, treatment with lorlatinib significantly reduced the tumor weight (Fig. 5b). However, we did not observe any further decrease in tumor weight when combining lorlatinib with gemcitabine compared to lorlatinib alone (Fig. 5b). We analyzed the immune cell composition of tumors from all treatment groups by flow cytometry and found reduced neutrophil accumulation in all treatment groups compared to the control group (Supplementary Fig. 9a). We did not see any difference in monocytes, macrophages, or CD8 T cells between any of the treatment groups (Supplementary Fig. 9b–d). The reduction in neutrophil accumulation was confirmed by IHC of tumor sections (Supplementary Fig. 9e). Finally, we analyzed proliferation, fibrosis, and fibroblast accumulation in tumors from all treatment groups by IHC and found no added benefit of adding gemcitabine to lorlatinib compared to lorlatinib alone (Supplementary Fig. 9f–h). These results support previous reports that gemcitabine treatment has little effect on PDAC and further suggest that there is no additive effect of combining lorlatinib with gemcitabine in PDAC.

To determine if lorlatinib could improve the response to immunotherapy in PDAC, we treated mice bearing orthotopic KPC tumors with anti-PD-1 alone or in combination with lorlatinib (5 mg/kg) (Fig. 5c). Consistent with previous reports^51^, we found that treatment with anti-PD-1 had no effect on the weight of orthotopic tumors compared to the control group (control IgG + vehicle). However, when combined with lorlatinib, anti-PD-1 resulted in significantly smaller tumors compared to tumors from the control group or single treatment groups (Fig. 5d). We confirmed the reduced accumulation of neutrophils in tumors from mice treated with lorlatinib alone or lorlatinib combined with anti-PD-1 by both flow cytometry and IHC staining of tumor sections (Fig. 5e, g). Interestingly, while treatment with anti-PD-1 or lorlatinib alone was insufficient at stimulating an increase in CD8^+^ T cells in tumors, combination treatment with lorlatinib and anti-PD-1 significantly increased the accumulation of CD8^+^ T cells as seen by both flow cytometry and IHC staining (Fig. 5f, g). In addition, combination treatment with lorlatinib and anti-PD-1 also resulted in more activated CD44^+^ CD69^+^ CD8^+^ T cells (Fig. 5h). Taken together, these data suggest that treatment with lorlatinib can make unresponsive PDAC tumors responsive to immunotherapy with anti-PD-1 blockade.

## Discussion

It is clear that improvements in treatment are urgently needed for patients suffering from PDAC, which is characterized by a dismal prognosis. PDAC tumors are characterized by a TME with excessive deposition of ECM and infiltration of different immune cells, which are believed to support tumor progression. At the same time, the ability to modulate cells within the TME might improve the therapeutic response of PDAC patients. In this study, we find that treatment with lorlatinib suppresses tumor growth in pre-clinical models of both PDAC and CRC. These models reflect growth of the primary tumor and metastases to the liver, respectively, and suggest that lorlatinib primarily targets tumor-associated neutrophils, since we did not observe any direct effect of lorlatinib on pancreatic cancer cells or CAFs in vitro. However, we cannot entirely exclude a direct in vivo effect of lorlatinib on other intratumoral processes. Lorlatinib was also able to extend the survival of a genetically engineered PDAC mouse model that expresses mutant alleles of *Kras* and *Tp53* and develop pancreatic tumors whose pathophysiological and molecular features resemble those of human PDAC. The results from these models indicate that lorlatinib has a systemic effect, independent of the tumor-affected organ since tumors in both the pancreas and metastases in the liver demonstrated reduced fibrosis and limited cancer cell proliferation.

Neutrophils have emerged as an influential component of the TME in different cancer types^15,52^, including PDAC, but despite recent advances in understanding neutrophil biology in cancer, the mechanisms responsible for the pathological activation of neutrophils are not well defined and this limits the selective targeting of these cells. Lorlatinib was originally developed to target ALK and several ALK inhibitors have already been approved for the treatment of patients with ALK-positive cancers^53,54^, including lorlatinib which was proven safe and tolerable in phase I and II trials^25,55^. While lorlatinib was designed to primarily target ALK, it can also inhibit the activity of the non-receptor tyrosine kinase FES^26^. In this study, we find that PDAC cells activate FES signaling in neutrophils and that lorlatinib can block this activation in vitro. Furthermore, we demonstrate that lorlatinib reduces tumor-induced granulopoiesis and neutrophil motility, which could have important implications for cancer patients since recent results suggest that immature neutrophil subsets, which are highly pro-tumorigenic, are produced in large numbers, released from the BM and further recruited into the TME^46,56–58^. It will be interesting to determine the precise role of FES kinase in neutrophil biology in future studies. In mouse models of PDAC, depletion of neutrophils using anti-Ly6G antibodies or inhibition of CXCR2 signaling results in reduced tumor growth, abrogation of metastasis, and improved influx of cytotoxic T cells, which enables an improved checkpoint inhibition therapy response^13,59,60^. Interestingly, one study demonstrated that neutrophil inhibition can lead to a compensatory increase in other myeloid cells in PDAC tumors, such as macrophages^5^, which has important implications when deciding a treatment strategy for cancer patients. Our results suggest that lorlatinib mainly affects neutrophils since we did not observe any consistent differences in the accumulation of macrophages or other immune cell types in our different models. Although, we cannot formally exclude the possibility that lorlatinib directly affects other immune cell types in our models in vivo. In contrast, our data suggest that lorlatinib rather suppresses neutrophil development in the BM and inhibits neutrophil-induced tumor growth. In this context, lorlatinib could be used as a therapeutic option to suppress the development and release of neutrophil subsets in cancer patients. It is important to note the heterogeneity of neutrophils in cancer, with no clear defining cell surface markers to distinguish tumor-associated neutrophils from myeloid-derived suppressor cells, which are defined as immature with highly immunosuppressive functions^61^. For that reason, we have consistently used the term neutrophils to identify CD11b^+^Ly6C^mid^Ly6G^+^ cells, but it would be interesting to determine the specific role of FES in regulating the functional phenotype of neutrophils.

In addition, it could be a promising approach in the future to validate the efficacy of lorlatinib in other cancer types with a high degree of neutrophil recruitment since lorlatinib has already been tested in humans, and approved for use in the clinic^25,55^.

In accordance with previous studies^6,51^, we did not observe any effect of gemcitabine on tumor weight in our study, and the combination of gemcitabine with lorlatinib did not enhance the therapeutic effect of gemcitabine compared to lorlatinib alone. However, one interesting aspect of that experiment is the observation that treatment with lorlatinib, but not gemcitabine, results in reduced fibrosis and aSMA^+^ fibroblast accumulation. Similarly, we observe reduced fibrosis and fibroblast accumulation in the tumor after neutrophil depletion, similar to our observations with lorlatinib treatment, but with no further reduction in fibrosis after neutrophil depletion combined with lorlatinib. These observations suggest that the reduction in fibrosis and activated fibroblasts, potentially through neutrophil-fibroblast crosstalk, may be an important mechanism by which lorlatinib reduces tumor growth. Finally, while we did not see an improved tumor response to the combination of lorlatinib and gemcitabine compared with lorlatinib alone, we demonstrate that lorlatinib can improve the therapeutic response to anti-PD-1 treatment leading to reduced tumor size compared with lorlatinib or anti-PD-1 treatments alone and increased accumulation and activation of CD8^+^ T cells, which would be important for PDAC patients. In summary, we demonstrate that lorlatinib indirectly suppresses the growth of PDAC at primary and metastatic sites by suppressing neutrophil development in the BM and modulating tumor-promoting neutrophil functions within the TME.

## Methods

### Animal studies

All experiments were carried out under authorization and guidance from the Danish Inspectorate for Animal Experimentation and UK Home Office license and approved by the University of Glasgow Animal Welfare and Ethical Review Board. *Kras*^*LSL-G12D/+*^*;Trp53*^*LSL-R172H/+*^*;Pdx1–Cre* mice were described previously^48^ and were bred in Glasgow on a mixed background. KPC mice were monitored at least three times weekly and culled when exhibiting symptoms of PDAC. Both male and female adult KPC mice were included in the study. Female C57BL/6 mice (age 6–12 weeks; Taconic, Denmark) were used for orthotopic or intrasplenic injections of KPC mT4 cells, whereas male C57BL/6 mice (6–12 weeks; Charles River, UK) were used for intrasplenic injections of *villin*CreER *Apc*^*fl/fl*^*Kras*^*G12D/+*^*Trp53*^*fl/R172H*^*TgfbrI*^*fl/fl*^ organoids. Cohorts of mice were randomized into different treatment groups before the start of treatment. At least four mice were included per group for each experiment.

### Tumor models and treatments

#### Orthotopic tumor model

Age-matched female C57BL/6 mice (6–12-weeks-old) were used for the orthotopic mouse model. Orthotopic PDAC tumors were established by surgical implantation. Briefly, 1 × 10^5^ KPC mT4 cells in 20 µL growth factor-reduced Matrigel were injected into each pancreas. Cohorts of mice were randomized into different treatment groups by gross tumor diameter using twice-weekly palpation. Tumor burden was measured by establishing the gross wet weight of the pancreas.

#### Experimental liver metastasis model

Experimental liver metastasis was performed by injecting 5 × 10^5^ KPC mT4 or CRC cells in PBS into the spleen of immunocompetent syngeneic female C57BL/6 mice using a Hamilton 29-G Syringe^12^. For experimental colorectal cancer liver metastasis, single-cell suspensions were prepared from *villin*CreER *Apc*^*fl/fl*^*Kras*^*G12D/+*^*Trp53*^*fl/R172H*^*TgfbrI*^*fl/fl*^ organoids before injecting into the spleen of immunocompetent syngeneic male C57BL/6 mice. At the indicated time points, mice were euthanized and metastatic tumor burden was assessed by the weight of the liver relative to the whole mouse. We further assessed the metastatic burden quantifying the frequency and size of metastatic lesions in hematoxylin and eosin-stained paraffin-embedded liver sections by microscopy using a Hamamatsu NanoZoom slide scanner and NDP.view2 software. The metastatic index was calculated as the accumulated size of all metastatic lesions as a percentage of the total area of the liver section.

#### Treatments

Lorlatinib was purchased from SelleckChem and dissolved in 2% DMSO, 30% polyethyleneglycol 300, and 68% Hank’s balanced salt solution (HBSS). Mice were given a daily oral administration of lorlatinib (5 mg/kg body weight) or vehicle control using sterile 20-G feeding needles.

For neutrophil depletion, mice were treated with 200 μg/dose of InVivoMAb anti-mouse Ly6G (clone 1A8; BioXCell, Cat # BP0075-1) or isotype control antibody (clone 2A3, BioXcell, Cat # BP0089) diluted in InVivoPure pH 7.0 Dilution Buffer (BioXcell, Cat # IP0070) via intraperitoneal injections three times a week for 2 weeks. Gemcitabine (Sigma, Cat # G6423) was dissolved in PBS, and mice were treated with 50 mg/kg by intraperitoneal injection two times a week for 2 weeks. For anti-PD-1 treatment, mice were treated with 250 μg/dose of InVivoMAb anti-mouse PD-1 (clone RMP1-14; BioXCell, Cat # BE0146) or isotype control antibody (clone 2A3, BioXcell, Cat # BP0089) diluted in InVivoPure pH 7.0 Dilution Buffer (BioXcell, Cat # IP0070) via intraperitoneal injections three times a week for 2 weeks.

### Cell culture

Murine pancreatic cancer cells KPC mT4, generated in the Tuveson Laboratory (Cold Spring Harbor Laboratory, NY, USA), were isolated from PDAC tumor tissues obtained from *Kras*^*LSL-G12D/+*^*;Trp53*^*LSL-R172H/+*^*;Pdx1–Cre* mice of a pure C57BL/6 background^62^. KPC mT4 cells were cultured in DMEM + 10% FBS and supplemented with 100 units of penicillin and 100 µg ml^−1^ streptomycin. For some experiments, we used KPC mT4 cells that were engineered to express the fluorescent protein zsGreen and Firefly Luciferase. These cells were generated by lentiviral particle infection with pHIV Luc–zsGreen (gift from B. Welm, University of Utah, USA, Addgene plasmid no. 39196). Cells were sorted by flow cytometry for high zsGreen expression levels. CAF cell lines 17964-56 and 19238-43, generated in the Tuveson laboratory^42^ (Cold Spring Harbor Laboratory, NY, USA), were isolated from PDAC tumor tissues obtained from *Kras*^*LSL-G12D/+*^*;Trp53*^*LSL-R172H/+*^*;Pdx1–Cre* mice and cultured in DMEM + 5% FBS and supplemented with 100 units of penicillin and 100 µg ml^−1^ streptomycin. Murine colorectal cancer cells were generated from tumors in *villin*CreER *Apc*^*fl/fl*^ *Kras*^*G12D/+*^ *Trp53*^*fl/R172H*^ *TgfbrI*^*fl/fl*^ mice^41^ and cultured as organoids in Matrigel (BD Biosciences, 356231) with Advanced DMEM/F12 supplemented with penicillin/streptomycin (100 U/ml / 100 µg/ml) (15140122), 2 mM l-glutamine (25030081), 10 mM HEPES (15630080), N2-supplement (17502001), and B27-supplement (17504044) (all ordered from Gibco, Life Technologies, ThermoFisher Scientific, Paisley, UK), 50 ng/ml Recombinant Human EGF (AF-100-15, Peprotech, London, UK), 100 ng/ml Recombinant Murine Noggin (250-38, Peprotech). All cells were cultured at 37 °C, 5% CO_2_ in a humidified chamber. All cell lines were routinely tested negative for mycoplasma and tested negative for murine pathogens by IMPACT testing (IDEXX Laboratories, USA). None of the cell lines used in this manuscript are listed in the ICLAC and NCBI Biosample database of misidentified cell lines.

### Neutrophil isolation and purification

Primary murine neutrophils were isolated by flushing the BM from the femur and tibia of C57BL/6 mice followed by double density-gradient centrifugation on histopaque (1077 and 1119 g/mL) (Sigma, 10771 and 11191) at 300×*g* for 25 min (without brakes). All cells were collected from the two interfaces and further purified using the Neutrophil Isolation Kit (Miltenyi) according to the manufacturer’s instructions. The purity of isolated neutrophils was determined by flow cytometry using anti-CD45, anti-Ly6G, and anti-CD11b antibodies.

### Conditioned medium preparation and cytokine array

Conditioned medium from KPC mT4 cells was generated according to the previous reports^63^. The medium was removed from 70% confluent KPC mT4 cells and cells were washed three times with PBS before the addition of serum-free medium. Cells were incubated for 18–24 h in serum-free medium and then collected and filtered through 0.45-µm filters before immediate use.

Cytokine array was performed using Proteome Profiler Mouse Cytokine Array Kit, Panel A (R&D, #ARY006) according to the manufacturer’s instruction. The membranes were developed for 10 min. Data acquisition was performed using an ImageQuant^TM^ LAS 400 instrument and images were analyzed using ImageJ.

### Kinase profiling

The neutrophil cell lysate was prepared by lysis with Mammalian Protein Extraction Reagent (M-PER^TM^) containing Halt^TM^ Phosphatase Inhibitor Cocktail (1:100) and Halt^TM^ Protease Inhibitor Cocktail (1:100) for 30 min at 4 °C on a rocker. Cell extracts were cleared by centrifugation for 10 min at 16,000×*g* at 4 °C, and protein concentrations were determined by Bradford assays (Pierce). Aliquots of 1 mg/ml were prepared and snap-frozen in liquid nitrogen. Measurements were performed on a PamChip Kinase Profiling Microarray System (PamStation 12, PamGene)^27^. Briefly, 5 µg of protein extract was used for the PTK array protocol (V1.9) using chips containing peptides (with known phosphorylation sites) immobilized in an array format on a porous membrane. The PTK array was processed in a single-step reaction. Cell extracts, ATP, and fluorescein isothiocyanate (FITC)-labeled pY20 antibody were incubated on the chip, and the phosphorylation of the individual Tyr peptides was followed by fluorescence detection in real time. Development of the FITC fluorescence signal was detected. Signal intensities were analyzed in BioNavigator software (PamGene) and expressed as log fold change. Prediction of kinases responsible for altered phosphorylation between conditions was analyzed using the upstream kinase analysis app (2018 version; PamGene) and was based on multiple kinase–substrate relationship databases. A full list of predicted kinases is supplied in Supplementary Data Files 1–5.

### Transwell migration

To evaluate neutrophil chemotaxis, KPC-conditioned medium or individual recombinant cytokines/chemokines were added to the lower well of a 24-well plate and freshly isolated neutrophils were seeded into the upper chamber of the insert on a 3-µm porous membrane. After 2 h, inserts were removed, and the inside was swabbed thoroughly with cotton tips and then fixed in methanol containing 0.05% crystal violet. The number of migrated neutrophils was counted by bright-field microscopy and presented as the number of cells/field of view or calculated relative to the control using the formula: (number of migrated neutrophils in treatment/ number of migrated neutrophils in control) × 100.

### CAF contraction

To assess force‐mediated matrix contraction, CAFs (derived from pancreatic tumors in the KPC mouse model:^42^ 19238 or 17564: 8 × 10^4^ cells) were embedded in 100 μl collagen I at a concentration of ~4 mg/ml and seeded in 96‐well plates^64^. Once the gel was set (1 h), cells were washed once in normal media for 1 h and then replaced with fresh media with or without lorlatinib. Gel contraction was monitored after 72 h by taking photographs of the gels. To obtain the gel contraction value, the relative diameters of the well and the gel were measured using ImageJ software, and the percentage of contraction was calculated using the formula 100 × (well diameter − gel diameter)/well diameter.

### Bone marrow colony formation

Colony-formation assays were performed according to the manufacturer’s protocol (StemCell Technologies). Briefly, cells were isolated from the BM, as described above, and were cultured in duplicate (30.000 cells per plate) in methylcellulose-based medium (MethoCult #3534; StemCell Technologies) containing recombinant cytokines (SCF, IL-3, and IL-6) in the presence of lorlatinib or DMSO for 3 days. After 3 days of incubation at 37 °C, the dishes were photographed, and the total colony numbers and types of colonies in plates were evaluated under a microscope, and colonies with >20 cells were scored.

### Proliferation and viability

#### Cancer cell and fibroblast proliferation

KPC mT4 cells or fibroblasts were seeded at a concentration of 2000 cells per well in a 96-well plate and allowed to adhere overnight. The next day, fresh cell culture medium was added to the cells supplemented with lorlatinib (1 or 10 µM) or vehicle control (DMSO). The cells were cultured for 2 days, and growth was measured by adding 20 µl CellTiter96 AQ_ueous_ One Solution Reagent to the cells. After 1 h at 37 °C in a humidified 5% CO_2_ atmosphere, the absorbance at 490 nm was recorded using an ELISA plate reader. The same measurement was performed immediately after the cell culture medium change to measure day 0.

#### Neutrophil viability

Neutrophils were prepared fresh from the BM of healthy mice as described in the methods and seeded at a concentration of 100.000 cells per well in poly-l-lysine-coated 96-well plates. Cells were allowed to attach for 1 h before the addition of DMEM ( + 5% FBS) supplemented with either 10 ng/mL GM-CSF or 40 ng/mL G-CSF, or KPC-CM ( + 5% FBS). Lorlatinib was added to final concentrations of 1 and 10 μM. DMSO was used as vehicle control at a 0.01% vol. Cells were cultured for two days and viability measured by adding CellTiter-Glo according to the manufacturer’s instructions and measuring the luminescence using an ELISA plate reader. The luminescence signal was normalized to the measurement of cells on day 0 (after seeding).

### Neutrophil-KPC co-cultures

For neutrophil-induced proliferation of KPC mT4-Luc/zsGreen cells, neutrophils were isolated from the BM of healthy, tumor-free mice as described in the “Methods” and seeded (600.000 cells per well) in poly-l-lysine (10 µg/mL)-coated tissue culture plates in the presence of KPC-CM and vehicle or lorlatinib. A small number of KPC mT4-Luc/zsGreen cells (2.000 per well) were seeded in the chamber alone or together with the neutrophils and cultured for 2 days. Cells were fixed with 4% PFA, permeabilized with 0.5% Triton X-100, and stained with DAPI to quantify the number of zsGreen+ KPC mT4 cells in the co-cultures.

### Immunoblots

Cell lysates were prepared from neutrophils stimulated with serum-free medium or KPC-CM in the presence or absence of 10 µM lorlatinib with M-PER^TM^ Mammalian Extraction Buffer containing Halt^TM^ Phosphatase Inhibitor Cocktail (1:100) and Halt^TM^ Protease Inhibitor Cocktail, EDTA (1:100). Protein lysates were resolved on NuPAGE 4–12% Bis-Tris gels (ThermoFisher Scientific, #17080971) and transferred to nitrocellulose membranes. Membranes were blocked in 5% milk for 1 h and incubated with primary antibodies overnight at 4 °C. The next day, membranes were washed with PBS + 0,1% Tween20 and incubated with appropriate HRP-conjugated secondary antibodies for 1 h. Immunoblots were visualized on an ImageQuant^TM^ LAS 400 instrument and images were analyzed using ImageJ. Vinculin or α-tubulin were used as sample integrity controls on separate blots (Supplementary Fig. 2). A list of the antibodies used for immunoblots in this paper can be found in Supplementary Table 2.

### Quantitative RT-PCR

Cells were lysed by either directly sorting them by FACS directly into RLT lysis buffer (containing β-mercaptoethanol) or direct lysis in their cell culture vessel. RNA was extracted using the RNeasy Mini Kit (Qiagen) following the manufacturer’s instructions. RNA concentration was measured using a NanoDrop. Reverse transcription of 500 ng of RNA was performed using iScript cDNA Synthesis Kit (BioRad) following the manufacturer’s instructions. Primers corresponding to mouse ribosomal protein lateral stalk subunit P0 (*Rplp0*) (forward: CATCATCAATGGGTACAAGCGC, reverse: CAGTAAGTGGGAAGGTGTACTC), *Acta2* (forward: ATCACCATCGGAAATGAACG; reverse: CTGGAAGGTGGACAGAGAGG), *Col1a* (forward: CGATGGATTCCCGTTCGAGT; reverse: GAGGCCTCGGTGGACATTAG) or *Fes* (Qiagen; Mm_Fes_1_SG QuantiTect Primer Assay). Quantitative RT-PCR was performed on a LightCycler 480 II (Roche). Relative expression levels were normalized to *Rplp0* expression according to the formula 2^−(CT for gene of interest − CT *Rplp0*)^.

### Immunostaining of frozen or paraffin-embedded tissue sections

#### Frozen tissue sections

Murine liver tissues were embedded in Optimal Cutting Temperature (OCT) medium and stored at −80 °C. Tissue sections were fixed in 4% paraformaldehyde, permeabilized with PBS + 0.1% Triton X-100, blocked with PBS + 8% normal goat serum, and incubated with primary antibodies. Next, tissue sections were washed in PBS, stained with secondary antibodies including DAPI (Life Technologies, 1 µg/ml), and mounted using Dako Fluorescent Mounting Medium. Goat anti-rat or goat anti-rabbit secondary antibodies conjugated to AlexaFluor488 and AlexaFluor555 were used (Abcam, 1:500). Antibodies used for immunostainings of tissue sections are listed in Supplementary Table 2. All tissue sections were imaged using a Leica SP8 confocal microscope and quantified using ImageJ software.

#### Immunohistochemistry of paraffin-embedded tissue slides

Mouse tumors were resected, fixed in 10% neutral buffered formalin overnight at 4 °C, and paraffin-embedded according to the standard protocol. Five-micrometer sections were processed for hematoxylin and eosin (H&E) per standard protocol. For immunohistochemistry staining, tissue sections were first deparaffinized in xylene and rehydrated in graded ethanols. Antigen retrieval was performed for 10 min at 95–98 °C in 10 mM sodium citrate buffer, pH 6.0, or 1 mM EDTA, pH 8.0. Then, endogenous peroxidase activity was quenched by 3% H_2_O_2_ for 10 min. Sections were blocked in T-PBS containing 0.1% Triton X-100 (Sigma) and 5% goat or donkey serum. Incubations with primary antibodies were performed overnight at 4 °C in a humidified chamber, followed with appropriate HRP-conjugated secondary antibodies (Dako, K4001, or K4003) at room temperature. ImmPACT DAB Kit (Vector Laboratories, SK-4105) was used to develop signals per the manufacturer’s instructions. Sections were counterstained with hematoxylin and mounted using DPX mounting medium (CellPath, SEA-1304-00A). Multiplex immunofluorescence staining of tumor slides was carried out similarly to immunohistochemistry staining, except that sections were blocked in PBS containing 0.1% Tween20, 5% goat serum, and subsequently incubated with primary antibodies overnight. The next day, slides were washed and incubated with Alexa Fluor-conjugated secondary antibodies including DAPI (1 µg/ml) for 1 h at room temperature and mounted using Dako Fluorescent Mounting Medium. Antibodies used for immunostainings of tissue sections are listed in Supplementary Table 2.

### Flow cytometry

Single-cell suspensions from murine tumors or hepatic metastases were prepared by mechanical and enzymatic disruption in Hanks balanced salt solution (HBSS) with 1 mg ml^−1^ collagenase P (Roche) using the mTDK1 program on a gentleMACS™ Dissociator (Miltenyi Biotec). Briefly, cells in suspension were centrifuged for 5 min at 1200 r.p.m., resuspended in HBSS, and filtered through a 500-µm polypropylene mesh (Spectrum Laboratories). The cell suspension was resuspended in 1 ml 0.05% trypsin and incubated at 37 °C for 5 min. Cells were filtered through a 70-µm cell strainer and resuspended in PBS + 1% BSA. Cells were blocked for 10 min on ice with FC Block (BD Pharmingen, Clone 2.4G2) and then stained with Sytox viability marker (Life Technologies) and fluorochrome-conjugated antibodies.

BM was prepared by flushing the femur and tibia from both hind legs of euthanized mice. Hematopoietic lineage discrimination of BM samples was performed using fluorochrome-conjugated antibodies. Blood was harvested from euthanized mice by cardiac puncture using EDTA-coated syringes and 25-G needles. Red blood cells were lysed using BD Pharm Lyse (BD Biosciences, 555899). Flow cytometry was performed on a FACSAria III (BD Biosciences). All antibodies used for flow cytometry are listed in Supplementary Table 1.

### Quantification of absolute numbers of cells per tissue using fluorescent beads

CountBright beads (CountBright Absolute Counting Beads; Invitrogen) were added to each single-cell suspension stained for flow cytometry. At least 1000 beads were acquired per tube to ensure accuracy for estimation of an absolute number of cells. For tumor and BM, the absolute number of cells is presented as cells per tumor or BM (tibia and femur from both hind legs).

### Statistical analysis

For studies comparing differences between two groups, we used unpaired Student’s *t* tests. For the Kaplan–Meier survival study we used the log-rank test. For studies comparing more than two groups, we used ANOVA with appropriate post hoc testing. Differences were considered significant when *P* < 0.05. Data are presented as box-and-whisker plots showing the median (line), mean (plus sign), 25th and 75th percentiles (box), and 5th and 95th percentiles (whiskers) or as bar graphs showing mean ± standard deviation.

### Reporting summary

Further information on research design is available in the Nature Research Reporting Summary linked to this article.

## Supplementary information

Supplementary Information

Descriptions of Additional Supplementary Files

Supplementary Data 1

Supplementary Data 2

Supplementary Data 3

Supplementary Data 4

Supplementary Data 5

Reporting Summary

## Data Availability

The transcriptome data used in this study are available in the GEO database under accession code: GSE109467 (bulk RNAseq from murine bone marrow neutrophil subsets) or BloodSpot database (http://servers.binf.ku.dk/bloodspot) with HemaExplorer dataset (bulk RNAseq from human bone marrow subsets). A full list of the predicted kinase activity including the detected level of phosphorylation for each bait peptide is provided as Supplementary Data 1–5. The remaining data are available within the Article, Supplementary Information, or available from the authors upon reasonable request. Source data are provided with this paper.

## Source data

Source Data
