# Supplementary material for: Suppression of tumor-associated neutrophils by lorlatinib attenuates pancreatic cancer growth and improves treatment with immune checkpoint blockade
Source: Nat Commun. 2021 Jun 7;12:3414. doi: 10.1038/s41467-021-23731-7 (PMC8184753; doi:10.1038/s41467-021-23731-7)
Supplement: Supplementary file 1 — Supplementary Information [file 41467_2021_23731_MOESM1_ESM.pdf]

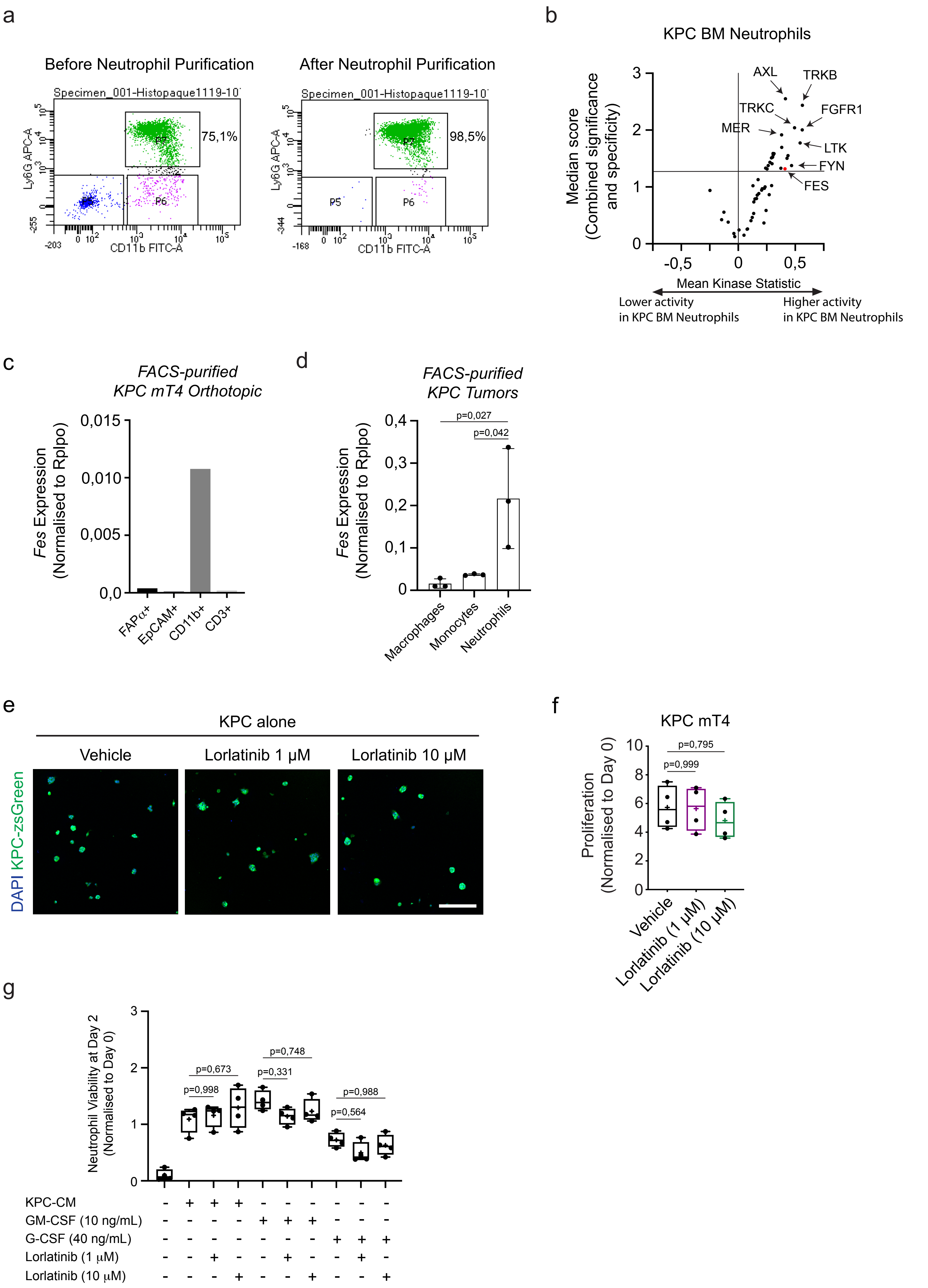
a
Before Neutrophil Purification
After Neutrophil Purification
Ly6G APC-A
CD11b FITC-A
75,1%
98,5%
P5
P6
P7
b
KPC BM Neutrophils
Median score
(Combined significance and specificity)
Mean Kinase Statistic
Lower activity in KPC BM Neutrophils
Higher activity in KPC BM Neutrophils
AXL
TRKB
TRKC
FGFR1
MER
LTK
FYN
FES
c
FACS-purified KPC mT4 Orthotopic
Fes Expression (Normalised to Rplpo)
FAPα+
EpCAM+
CD11b+
CD3+
d
FACS-purified KPC Tumors
Fes Expression (Normalised to Rplpo)
p=0,027
p=0,042
Macrophages
Monocytes
Neutrophils
e
KPC alone
Vehicle
Lorlatinib 1 μM
Lorlatinib 10 μM
DAPI KPC-zsGreen
f
KPC mT4
Proliferation (Normalised to Day 0)
p=0,795
p=0,999
Vehicle
Lorlatinib (1 μM)
Lorlatinib (10 μM)
g
Neutrophil Viability at Day 2 (Normalised to Day 0)
p=0,673
p=0,998
p=0,748
p=0,331
p=0,988
p=0,564
KPC-CM
GM-CSF (10 ng/mL)
G-CSF (40 ng/mL)
Lorlatinib (1 μM)
Lorlatinib (10 μM)

Supplementary figure 1. *Fes* is mainly expressed by neutrophils in the pancreatic tumor microenvironment. (a) Representative flow cytometry staining of BM-derived neutrophils before and after purification with magnetic beads. Purity of the neutrophil isolation was assessed by staining with antibodies against CD45, CD11b and Ly6G (n = 2 mice; representative of 1 independent experiment). 98,5% of recovered viable ($SYTOX^{neg}$) cells were $CD45^+$ $CD11b^+$ $Ly6G^+$ neutrophils after purification with magnetic beads. (b) Volcano plots of the kinase activity prediction based on bait peptide phosphorylation of PTK chip arrays with protein lysate from BM neutrophils isolated from tumor-bearing KPC mice or tumor-free control mice (data show results with three independent samples from each group). (c) Relative expression of *Fes* by qPCR in FACS-sorted cell populations from an orthotopic PDAC tumor ($FAP\alpha^+$ fibroblasts, $EpCAM^+$ epithelial cells, $CD11b^+$ myeloid cells or $CD3^+$ T cells) (cells were sorted from 1 tumor). (d) Relative expression of *Fes* by qPCR in FACS-sorted cell populations from PDAC tumors in the KPC mouse model ($CD11b^+F4/80^+$ macrophages, $CD11b^+$ $F4/80^{neg}$ $Ly6C^+$ $Ly6G^{neg}$ monocytes or $CD11b^+$ $F4/80^{neg}$ $Ly6C^{low}$ $Ly6G^+$ neutrophils) (n = 3 mice, data are from one experiment). (e) Representative immunofluorescence images and quantification of KPC-zsGreen cells without neutrophils in the presence of vehicle or lorlatinib in 2 days of culture. Quantification is shown in Fig. 1e. Five FOV were acquired per condition from each experiment and used for quantification of $zsGreen^+$ cells (n = 3 independent experiments). (f) Quantification of proliferation of KPC mT4 cells in the presence of lorlatinib or vehicle for two days. The relative proliferation was normalized to the measurement of cells seeded on the same plates before treatment with vehicle or lorlatinib (n = 4 independent experiments). (g) Quantification of neutrophil viability after 2 days of stimulation with KPC-CM, 10 ng/ml GM-CSF or 40 ng/mL G-CSF in the presence of vehicle or lorlatinib (n = 4 independent experiments)). Scale bar represents 200µm. Box-and-whisker plot shows the median (line), mean (plus sign), 25th and 75th percentiles (box) and 5th and 95th percentiles (whiskers); bar graphs represent mean and standard deviation; hypothesis testing performed using two-way ANOVA with (d, f) Bonferroni's or (g) Sidak's method for multiple comparisons.

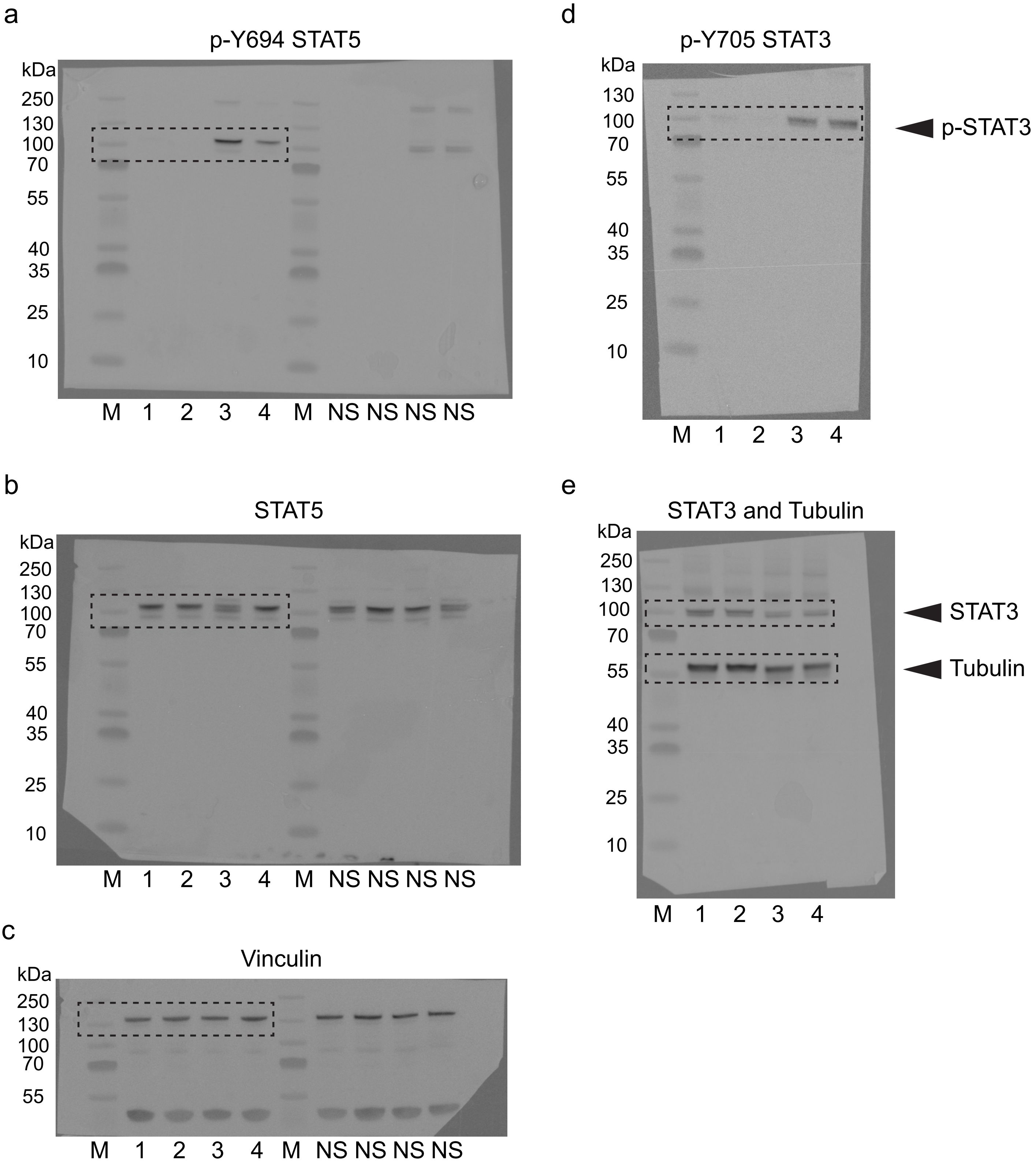
a
p-Y694 STAT5
kDa
250
130
100
70
55
40
35
25
10
M 1 2 3 4 M NS NS NS NS
b
STAT5
kDa
250
130
100
70
55
40
35
25
10
M 1 2 3 4 M NS NS NS NS
c
Vinculin
kDa
250
130
100
70
55
M 1 2 3 4 M NS NS NS NS
d
p-Y705 STAT3
kDa
130
100
70
55
40
35
25
10
M 1 2 3 4
p-STAT3
e
STAT3 and Tubulin
kDa
250
130
100
70
55
40
35
25
10
M 1 2 3 4
STAT3
Tubulin

Supplementary figure 2. Full scans of immunoblots from figure 1c. Dashed boxes indicate cropped regions displayed in Figure 1c. Arrows indicate the expected band position for each protein or phosphorylation site probed. M, molecular weight markers; NS, non-relevant sample; 1, Neutrophils treated with control medium and vehicle for 1 hour; 2, Neutrophils treated with control medium and 10μM lorlatinib for 1 hour; 3, Neutrophils treated with KPC-conditioned medium and vehicle for 1 hour; 4, Neutrophils treated with KPC-conditioned medium and 10μM lorlatinib for 1 hour.

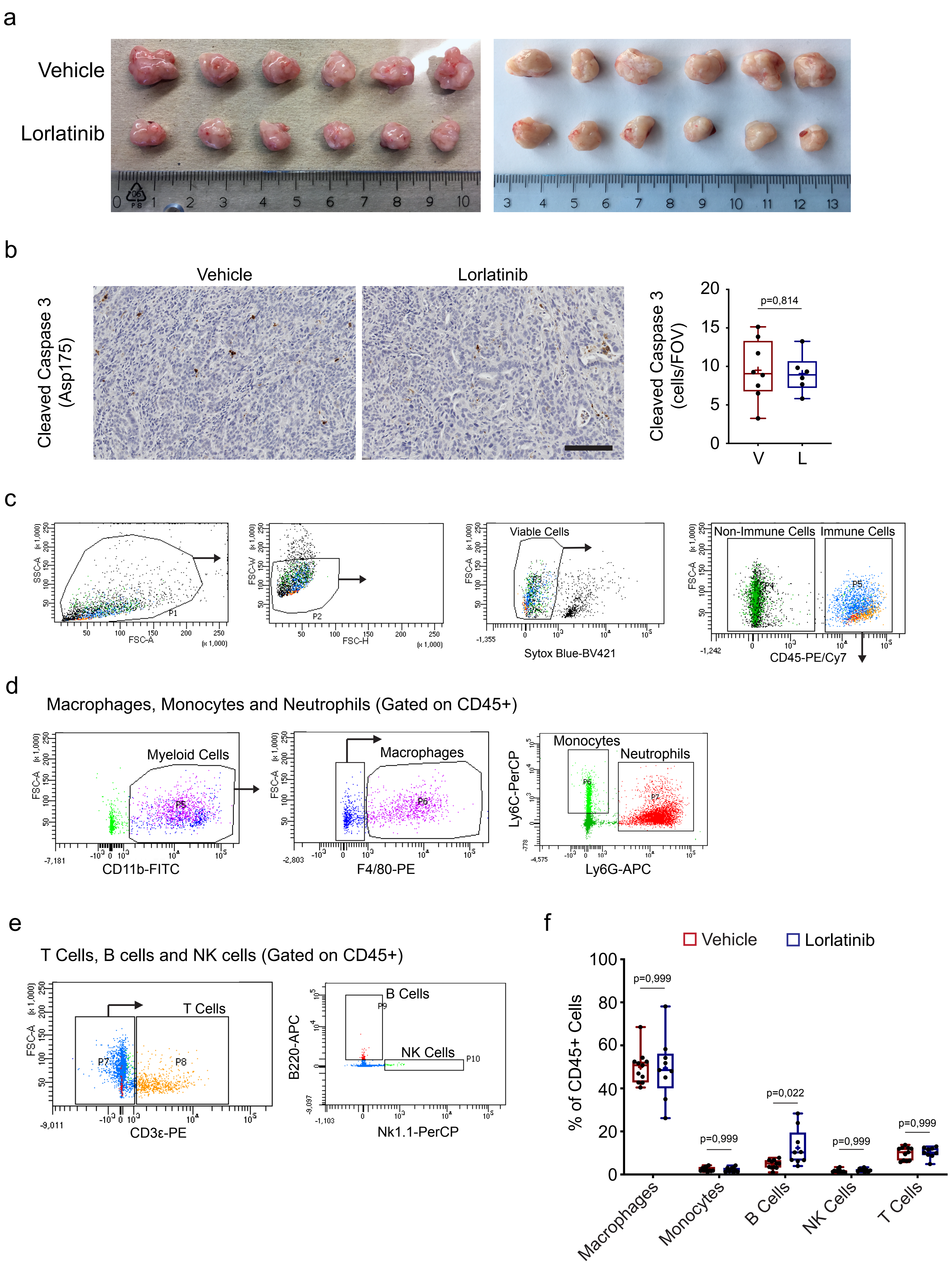
a
Vehicle
Lorlatinib
b
Vehicle
Lorlatinib
Cleaved Caspase 3 (Asp175)
Cleaved Caspase 3 (cells/FOV)
p=0,814
V
L
c
Viable Cells
Non-Immune Cells
Immune Cells
FSC-A
SSC-A
FSC-H
FSC-W
Sytox Blue-BV421
CD45-PE/Cy7
d
Macrophages, Monocytes and Neutrophils (Gated on CD45+)
Myeloid Cells
Macrophages
Monocytes
Neutrophils
CD11b-FITC
F4/80-PE
Ly6C-PerCP
Ly6G-APC
e
T Cells, B cells and NK cells (Gated on CD45+)
T Cells
B Cells
NK Cells
CD3ε-PE
B220-APC
Nk1.1-PerCP
f
Vehicle
Lorlatinib
% of CD45+ Cells
p=0,999
p=0,999
p=0,022
p=0,999
p=0,999
Macrophages
Monocytes
B Cells
NK Cells
T Cells

Supplementary figure 3. Lorlatinib treatment attenuates PDAC tumor progression. (a) Representative photographs of orthotopic PDAC tumors at day 28 from vehicle- or lorlatinib-treated mice. Images are from two independent experiments. (b) Representative IHC images and quantification of cleaved caspase 3 (Asp175)-positive cells from in orthotopic PDAC tumors from vehicle- and lorlatinib-treated mice (n = 8 mice, vehicle; n = 6 mice, lorlatinib; data are from two experiments). Six images were acquired per mouse and used for quantification of cleaved caspase 3^{+} cells. (c-e) Representative flow cytometry gating strategy for quantification of the cellular composition of the TME from orthotopic PDAC tumors. (c) Representative flow cytometry dot plots showing the gating strategy used to identify hematopoietic cells ($CD45^{+}$). Only viable ($SYTOX^{neg}$) cells were used for analysis. (d) Representative flow cytometry dot plots showing gating strategy used to identify macrophages ($CD11b^{+}F4/80^{+}$), neutrophils ($CD11b^{+}F4/80^{neg}Ly6G^{+}$) and inflammatory monocytes ($CD11b^{+}F4/80^{neg}Ly6G^{neg}Ly6C^{+}$) from the hematopoietic cell population ($CD45^{+}$). (e) Representative flow cytometry dot plots showing gating strategy used to identify B cells ($B220^{+}$), T cells ($CD3^{+}$) and NK cells ($CD3^{neg}B220^{neg}$ $NK1.1^{+}$) from hematopoietic cells ($CD45^{+}$). (f) Flow cytometry analysis of immune cells in PDAC tissue from mice bearing orthotopic tumors treated for 14 days with vehicle or lorlatinib. The composition of intratumoural immune cells is shown as a percentage of $CD45^{+}$ cells (n = 11 mice, vehicle; n = 9 mice, lorlatinib; data are from two experiments). Scale bar represents 100µm (b). Box-and-whisker plot shows the median (line), mean (plus sign), 25th and 75th percentiles (box) and 5th and 95th percentiles (whiskers); hypothesis testing performed using unpaired two-sided Student's t test (b) or two-way ANOVA with Tukey's method for multiple comparisons (f).

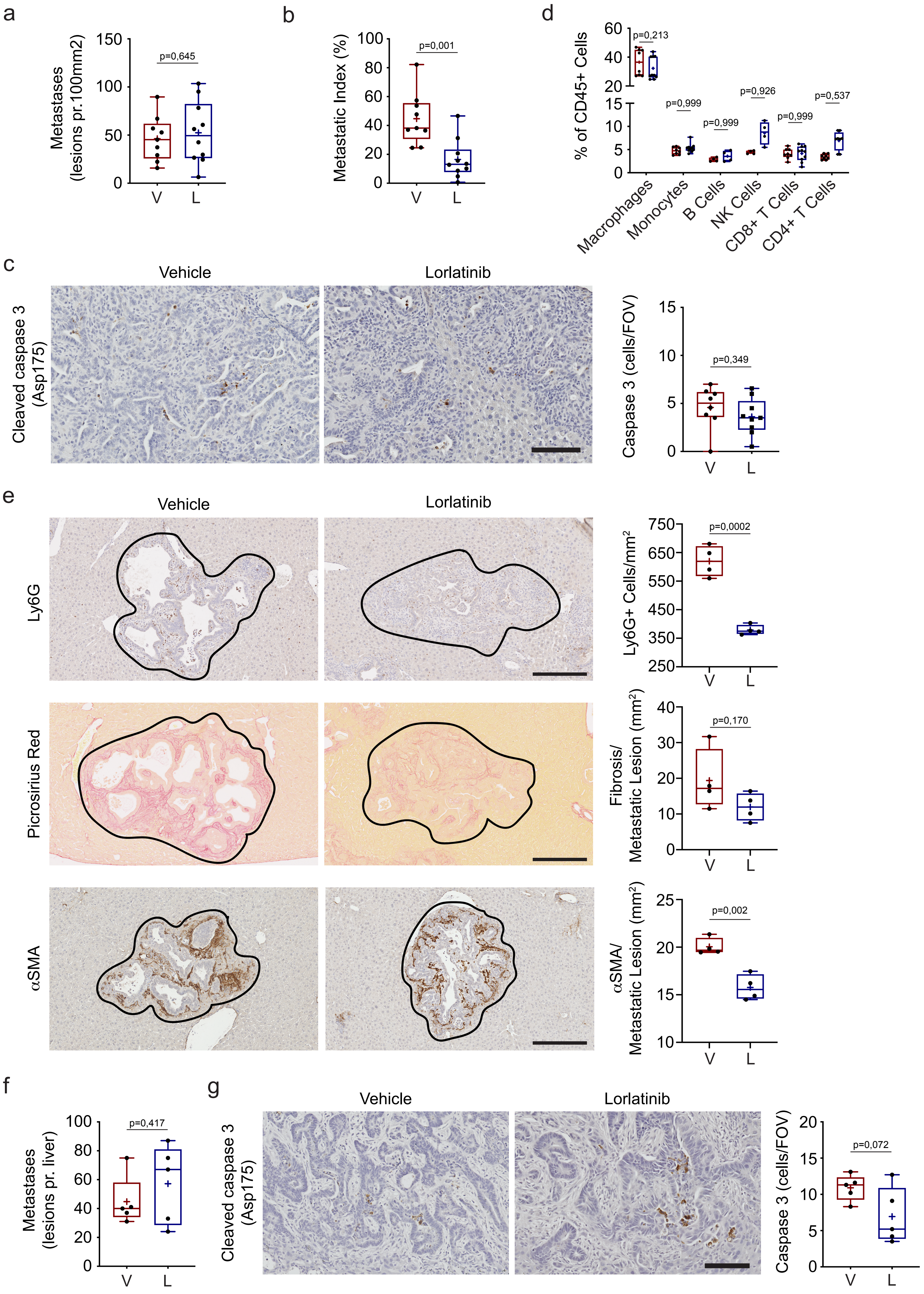

a
Metastases (lesions pr.100mm2)
150
100
50
0
p=0,645
V
L
b
Metastatic Index (%)
100
80
60
40
20
0
p=0,001
V
L
d
% of CD45+ Cells
60
40
20
15
10
5
0
p=0,213
p=0,999
p=0,999
p=0,926
p=0,999
p=0,537
Macrophages
Monocytes
B Cells
NK Cells
CD8+ T Cells
CD4+ T Cells
c
Vehicle
Lorlatinib
Cleaved caspase 3 (Asp175)
Caspase 3 (cells/FOV)
15
10
5
0
p=0,349
V
L
e
Vehicle
Lorlatinib
Ly6G
Picrosirius Red
αSMA
Ly6G+ Cells/mm2
750
650
550
450
350
250
p=0,0002
V
L
Fibrosis/ Metastatic Lesion (mm2)
40
30
20
10
0
p=0,170
V
L
αSMA/ Metastatic Lesion (mm2)
25
20
15
10
p=0,002
V
L
f
Metastases (lesions pr. liver)
100
80
60
40
20
0
p=0,417
V
L
g
Vehicle
Lorlatinib
Cleaved caspase 3 (Asp175)
Caspase 3 (cells/FOV)
20
15
10
5
0
p=0,072
V
L

Supplementary figure 4. Lorlatinib reduces the hepatic metastatic burden in mouse models of pancreatic and colorectal cancer metastasis. Hepatic metastatic burden three weeks after intrasplenic injection of KPC mT4 cells. Mice received daily treatment with vehicle or lorlatinib (5mg/kg) starting 7 days after injection for further 14 days. Metastatic burden was determined three weeks after injection by hematoxylin and eosin staining. (a) Number of hepatic metastatic lesions per 100 mm^2 (n = 9 mice, vehicle; n = 10 mice, lorlatinib; data are from two independent experiments). (b) Metastatic index calculated as the percentage of metastatic lesions that covers each liver section (n = 9 mice, vehicle; n = 10 mice, lorlatinib; data are from two independent experiments). (c) Representative images of cleaved caspase 3 (Asp175) IHC images of hepatic metastatic tissue from intrasplenic injection of KPC mT4 cells (n = 8 mice, vehicle; n = 9 mice, lorlatinib; data are from two experiments). (d) Flow cytometry analysis of immune cells in hepatic metastases shown as a percentage of $CD45^+$ cells (n = 8 mice, vehicle; n = 10 mice, lorlatinib; data are from two independent experiments except B and NK cells where n = 4 mice per condition from one experiment). (e) Representative images and quantification of IHC staining for neutrophils (Ly6G), fibrosis (picrosirius red) and fibroblasts (aSMA) in size-matched hepatic metastases (n = 4 mice per condition; data are from one experiment). Eight images were acquired per IHC staining per mouse and used for quantification as a percentage of the total area. (f) Number of metastatic lesions per liver after intrasplenic injection of CRC cells followed by treatment with vehicle control or lorlatinib (n = 5 mice per group; data are from one experiment). (g) Representative cleaved caspase 3 (Asp175) IHC images of hepatic metastatic tissue from intrasplenic injection of CRC organoids followed by treatment with vehicle control or lorlatinib (n = 5 mice per condition; data are from one experiment). Ten images were acquired per mouse and used for quantification of cleaved caspase 3^+ cells. Scale bar represents 100 μm (c, g) or 250 μm (e). Box-and-whisker plot shows the median (line), mean (plus sign), 25th and 75th percentiles (box) and 5th and 95th percentiles (whiskers); hypothesis testing performed using unpaired two-sided Student's t test (a-c, f-g) or two-way ANOVA with Tukey's method for multiple comparisons (d).

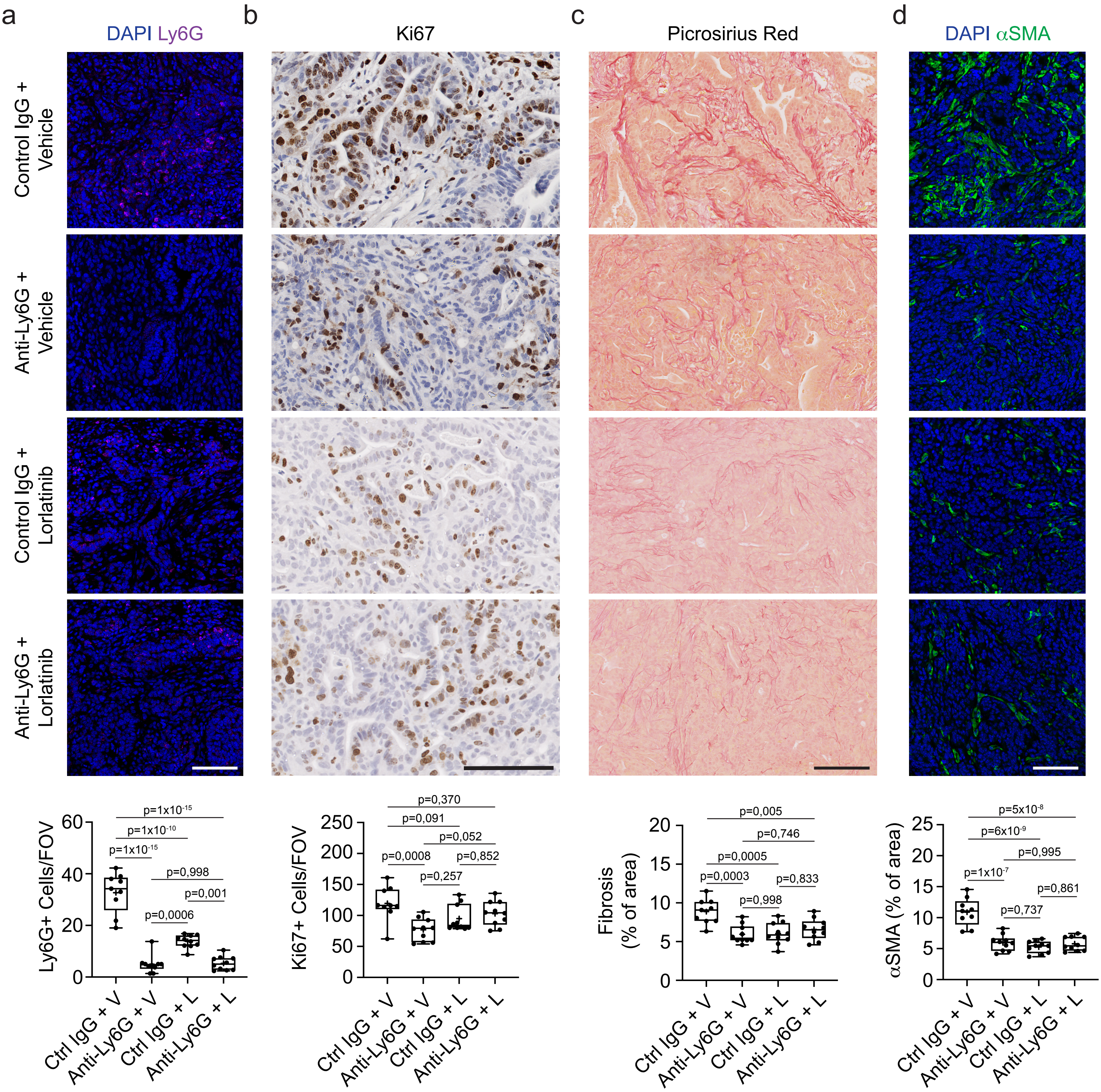

a
DAPI Ly6G
b
Ki67
c
Picrosirius Red
d
DAPI αSMA
Control IgG + Vehicle
Anti-Ly6G + Vehicle
Control IgG + Lorlatinib
Anti-Ly6G + Lorlatinib
Ly6G+ Cells/FOV
p=1x10-15
p=1x10-10
p=1x10-15
p=0,998
p=0,001
p=0,0006
Ki67+ Cells/FOV
p=0,370
p=0,091
p=0,052
p=0,0008
p=0,852
p=0,257
Fibrosis (% of area)
p=0,005
p=0,746
p=0,0005
p=0,0003
p=0,833
p=0,998
αSMA (% of area)
p=5x10-8
p=6x10-9
p=0,995
p=1x10-7
p=0,861
p=0,737
Ctrl IgG + V
Anti-Ly6G + V
Ctrl IgG + L
Anti-Ly6G + L

Supplementary figure 5. (a-d) Representative images and quantification of (a) neutrophils (Ly6G$^+$), (b) Ki67$^+$ cells, (c) fibrosis (picrosirius red) and (d) fibroblasts (αSMA$^+$) in tumors from mice treated with a combination of vehicle or lorlatinib and control IgG or anti-Ly6G. Five (Ly6G and aSMA) or eight (Ki67 and picrosirius red) images were acquired per mouse and used for quantifications (n = 9 mice, vehicle + control IgG; n = 10 mice, all other conditions; data are from two experiments). Scale bar represents 100 μm (a, b, d) or 250 μm (c). Box-and-whisker plot shows the median (line), mean (plus sign), 25th and 75th percentiles (box) and 5th and 95th percentiles (whiskers); hypothesis testing performed using two-way ANOVA with Tukey's method for multiple comparisons.

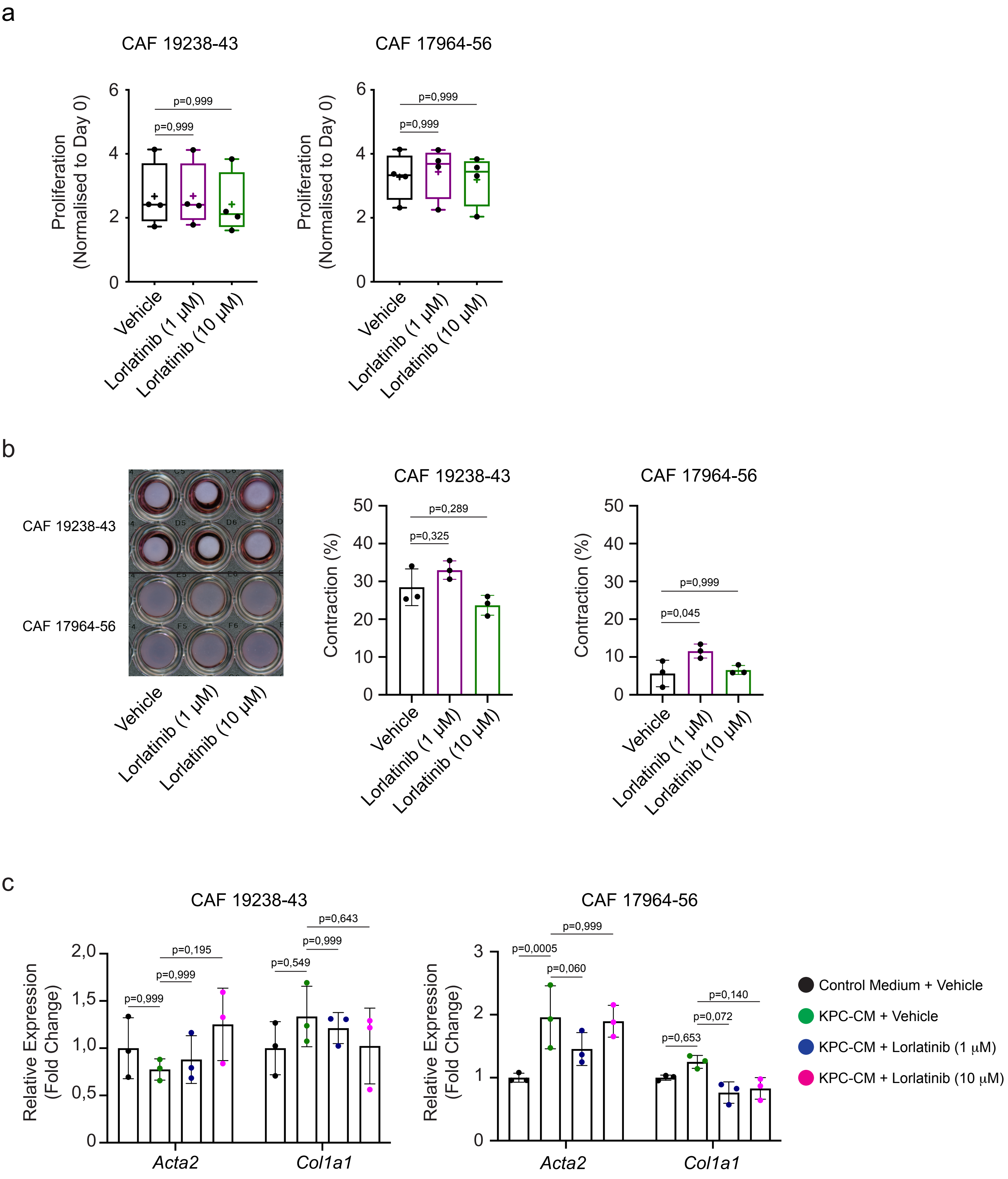

a
CAF 19238-43
CAF 17964-56
Proliferation (Normalised to Day 0)
p=0,999
p=0,999
Vehicle
Lorlatinib (1 µM)
Lorlatinib (10 µM)
b
CAF 19238-43
CAF 17964-56
Contraction (%)
p=0,289
p=0,325
p=0,999
p=0,045
Vehicle
Lorlatinib (1 µM)
Lorlatinib (10 µM)
c
CAF 19238-43
CAF 17964-56
Relative Expression (Fold Change)
p=0,999
p=0,999
p=0,195
p=0,549
p=0,999
p=0,643
p=0,0005
p=0,060
p=0,999
p=0,653
p=0,072
p=0,140
Acta2
Col1a1
Control Medium + Vehicle
KPC-CM + Vehicle
KPC-CM + Lorlatinib (1 µM)
KPC-CM + Lorlatinib (10 µM)

Supplementary figure 6. Lorlatinib does not inhibit the proliferation or activity of cancer-associated fibroblasts (CAFs). (a) Quantification of proliferation of KPC-derived CAF cell lines 19238-43 and 17964-56 in the presence of lorlatinib or vehicle for two days. The relative proliferation was normalized to the measurement of cells seeded on the same plates before treatment with vehicle or lorlatinib (n = 4 independent experiments). (b) Representative images and quantification of CAF-mediated force-contraction of extracellular matrix gels consisting 4 mg/ml collagen I. CAFs were embedded in gels in the presence of lorlatinib or vehicle and analyzed after three days (n = 3 independent experiments). (c) Quantification of *Acta2* and *Col1a1* expression by qPCR in CAFs stimulated with KPC-CM in the presence of vehicle or lorlatinib (n = 3 independent experiments). Box-and-whisker plot shows the median (line), mean (plus sign), 25th and 75th percentiles (box) and 5th and 95th percentiles (whiskers); bar charts show mean and standard deviation from independent experiments with each dot representing the mean of six technical replicates from 1 experiment; hypothesis testing performed using two-way ANOVA with Bonferroni's method for multiple comparisons.

a

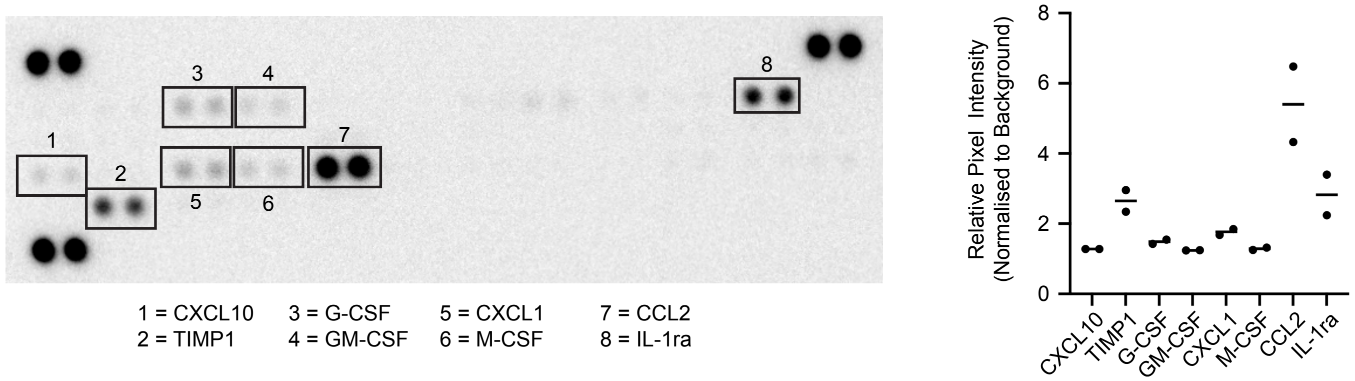
1
2
3
4
5
6
7
8
1 = CXCL10
2 = TIMP1
3 = G-CSF
4 = GM-CSF
5 = CXCL1
6 = M-CSF
7 = CCL2
8 = IL-1ra
Relative Pixel Intensity (Normalised to Background)
0
2
4
6
8
CXCL10
TIMP1
G-CSF
GM-CSF
CXCL1
M-CSF
CCL2
IL-1ra

b

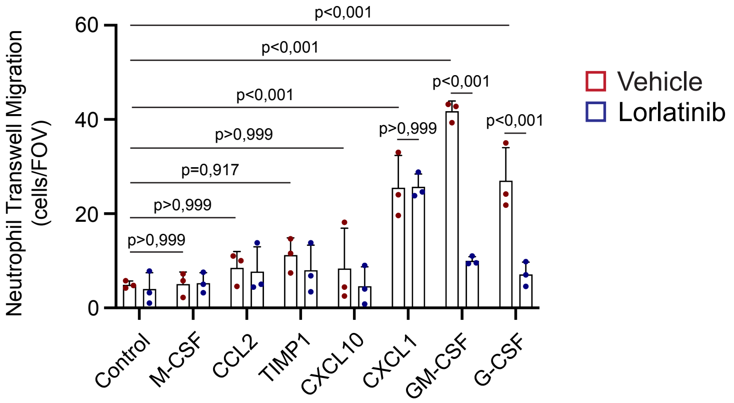
Neutrophil Transwell Migration (cells/FOV)
0
20
40
60
p<0,001
p<0,001
p<0,001
p>0,999
p=0,917
p>0,999
p>0,999
p>0,999
p<0,001
p<0,001
Vehicle
Lorlatinib
Control
M-CSF
CCL2
TIMP1
CXCL10
CXCL1
GM-CSF
G-CSF

Supplementary figure 7. Lorlatinib suppresses neutrophil migration in response to G-CSF and GM-CSF. (a) Representative image of the cytokine array and quantification of cytokines in conditioned medium from KPC mT4 cells (n=2 independent experiments). (b) Transwell migration of neutrophils towards recombinant cytokines and chemokines identified in (a) in the presence of vehicle or 1μM lorlatinib. Neutrophils were stimulated with 10ng/ml of CXCL10, G-CSF GM-CSF, M-CSF or CCL2, 20ng/mL of CXCL1 or 100ng/mL of TIMP-1 (n = 3 independent experiments). Lines show mean; Bar charts show mean and standard deviation from independent experiments with each dot representing the mean of five field of views (FOVs) from each experiment; hypothesis testing performed using two-way ANOVA with Bonferroni's method for multiple comparisons.

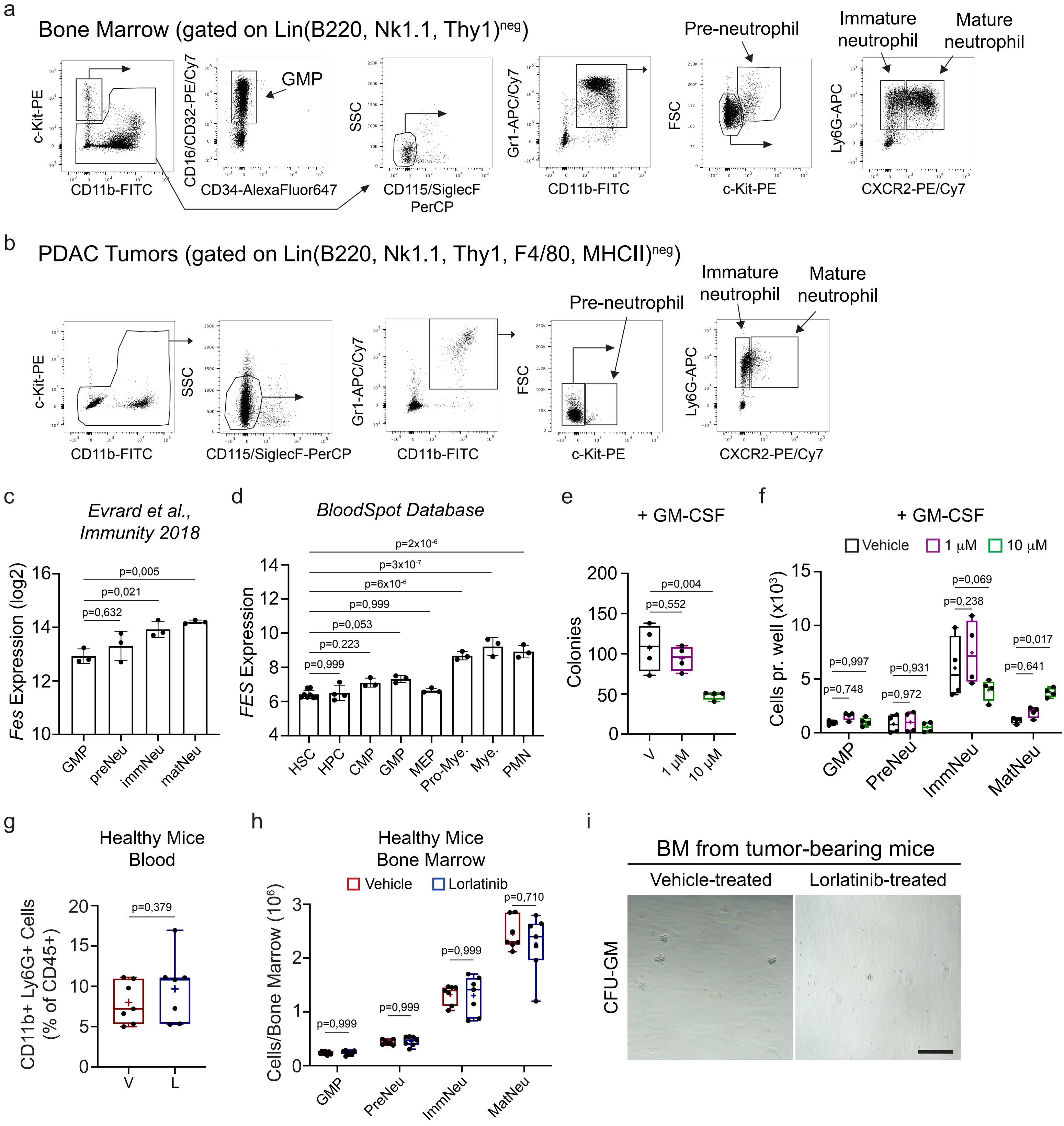
a
Bone Marrow (gated on Lin(B220, Nk1.1, Thy1)neg)
c-Kit-PE
CD11b-FITC
CD16/CD32-PE/Cy7
GMP
CD34-AlexaFluor647
SSC
CD115/SiglecF PerCP
Gr1-APC/Cy7
CD11b-FITC
Pre-neutrophil
FSC
c-Kit-PE
Immature neutrophil
Mature neutrophil
Ly6G-APC
CXCR2-PE/Cy7
b
PDAC Tumors (gated on Lin(B220, Nk1.1, Thy1, F4/80, MHCII)neg)
c-Kit-PE
CD11b-FITC
SSC
CD115/SiglecF-PerCP
Gr1-APC/Cy7
CD11b-FITC
Pre-neutrophil
FSC
c-Kit-PE
Immature neutrophil
Mature neutrophil
Ly6G-APC
CXCR2-PE/Cy7
c
Evrard et al., Immunity 2018
Fes Expression (log2)
p=0,005
p=0,021
p=0,632
GMP
preNeu
immNeu
matNeu
d
BloodSpot Database
FES Expression
p=2x10-6
p=3x10-7
p=6x10-6
p=0,999
p=0,053
p=0,223
p=0,999
HSC
HPC
CMP
GMP
MEP
Pro-Mye.
Mye.
PMN
e
+ GM-CSF
Colonies
p=0,004
p=0,552
V
1 μM
10 μM
f
+ GM-CSF
Vehicle
1 μM
10 μM
Cells pr. well (x10³)
p=0,997
p=0,748
p=0,931
p=0,972
p=0,069
p=0,238
p=0,017
p=0,641
GMP
PreNeu
ImmNeu
MatNeu
g
Healthy Mice Blood
CD11b+ Ly6G+ Cells (% of CD45+)
p=0,379
V
L
h
Healthy Mice Bone Marrow
Vehicle
Lorlatinib
Cells/Bone Marrow (10⁶)
p=0,710
p=0,999
p=0,999
p=0,999
GMP
PreNeu
ImmNeu
MatNeu
i
BM from tumor-bearing mice
Vehicle-treated
Lorlatinib-treated
CFU-GM

Supplementary figure 8. Lorlatinib suppresses neutrophil development. (a) Representative flow cytometry gating strategy for granulocyte-macrophage progenitors (GMP) and neutrophil subsets (pre-, immature and mature neutrophils) in the BM of tumor-bearing mice treated with vehicle or lorlatinib. (b) Representative gating strategy of neutrophil subsets in PDAC tumors from mice treated with vehicle or lorlatinib. (c) *Fes* expression in FACS-sorted murine BM-derived GMPs or neutrophil subsets from Evrard et al., Immunity 2018 (ref.1) (n = 3 mice per group). (d) *FES* expression in subsets of human hematopoietic cells from the BM of healthy donors in the BloodSpot database (ref. 2) (n = 8 biologically independent samples, HSC; n = 4 biologically independent samples, HPC; n = 3 biologically independent samples per group, remaining groups). Cell populations are labelled. HSC, hematopoietic stem cells; HPC, hematopoietic progenitor cells; CMP, common myeloid progenitor; GMP, granulocyte-monocyte progenitor; MEP, megakaryocyte–erythroid progenitor; pro-Mye., promyelocyte; Mye., myelocyte; PMN, polymorphonuclear neutrophils. (e, f) Number of colonies from CFU-GM assay (e) and flow cytometry analysis (f) of neutrophils after culture in semi-solid methylcellulose medium containing recombinant cytokines (SCF, IL-3 and IL-6) for 3 days in the presence of recombinant GM-CSF and lorlatinib or vehicle (n = 4 independent experiments). (g) Flow cytometry analysis of neutrophils ($CD45^+ CD11b^+ Ly6G^+$) in blood samples from healthy (tumor-free) mice treated with lorlatinib or vehicle for 14 days (n = 7 mice per group; data are from two independent experiments). (h) Flow cytometry analysis of GMPs and neutrophil subsets in BM from healthy (tumor-free) mice treated for 14 days with lorlatinib or vehicle (n = 7 mice per group; data are from two independent experiments). (i) Representative images of *colony forming unit-granulocyte macrophage* (CFU-GM) assay (from Fig. 3k) with BM cells isolated from mice with orthotopic PDAC tumors after treatment with vehicle or lorlatinib. Scale bar represents 200 µm. Box-and-whisker plot shows the median (line), mean (plus sign), 25th and 75th percentiles (box) and 5th and 95th percentiles (whiskers); bar charts show mean and standard deviation from independent biological replicates; hypothesis testing performed using unpaired two-sided Student's t test (g) or two-way ANOVA with Tukey's (c,e,f) or Sidak's (d) method for multiple comparisons.

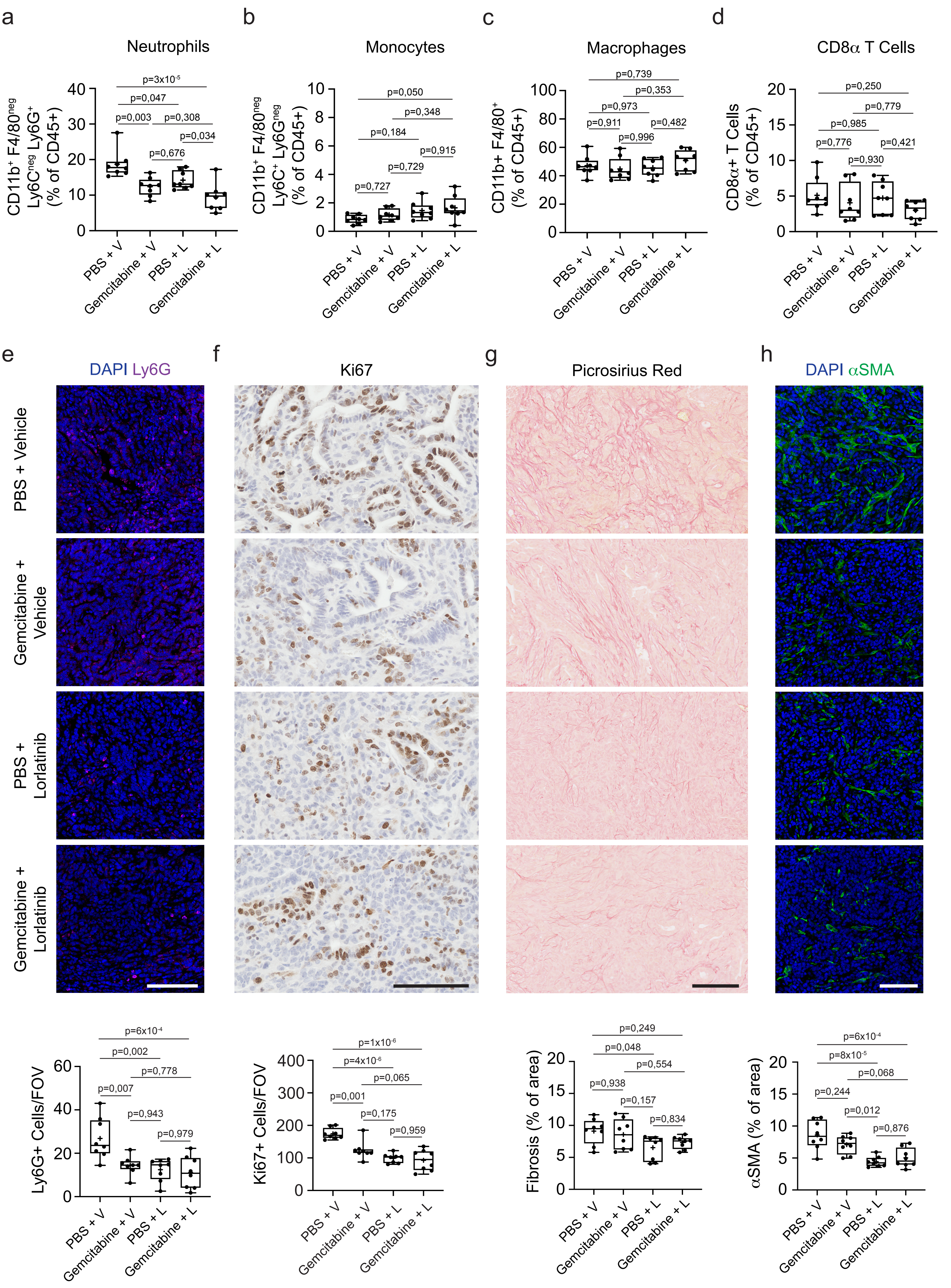
a
Neutrophils
CD11b+ F4/80neg Ly6Cneg Ly6G+ (% of CD45+)
p=3x10-5
p=0,047
p=0,003
p=0,308
p=0,034
p=0,676
b
Monocytes
CD11b+ F4/80neg Ly6C+ Ly6Gneg (% of CD45+)
p=0,050
p=0,348
p=0,184
p=0,915
p=0,729
p=0,727
c
Macrophages
CD11b+ F4/80+ (% of CD45+)
p=0,739
p=0,353
p=0,973
p=0,911
p=0,482
p=0,996
d
CD8α T Cells
CD8α+ T Cells (% of CD45+)
p=0,250
p=0,779
p=0,985
p=0,776
p=0,421
p=0,930
PBS + V
Gemcitabine + V
PBS + L
Gemcitabine + L
e
DAPI Ly6G
f
Ki67
g
Picrosirius Red
h
DAPI αSMA
PBS + Vehicle
Gemcitabine + Vehicle
PBS + Lorlatinib
Gemcitabine + Lorlatinib
Ly6G+ Cells/FOV
p=6x10-4
p=0,002
p=0,778
p=0,007
p=0,943
p=0,979
Ki67+ Cells/FOV
p=1x10-6
p=4x10-6
p=0,065
p=0,001
p=0,175
p=0,959
Fibrosis (% of area)
p=0,249
p=0,048
p=0,554
p=0,938
p=0,157
p=0,834
αSMA (% of area)
p=6x10-4
p=8x10-5
p=0,068
p=0,244
p=0,012
p=0,876

Supplementary figure 9. Combination of lorlatinib with gemcitabine does not further improve the therapeutic response to chemotherapy. (a-d) Flow cytometry analysis of (a) neutrophils, (b) monocytes, (c) macrophages or (d) CD8+ T cells in tumors from mice treated with a combination of vehicle or lorlatinib and PBS or gemcitabine (n = 8 mice per condition; data are from two independent experiments). (e-h) Representative images and quantification of (e) neutrophils ($Ly6G^+$), (f) $Ki67^+$ cells, (g) fibrosis (picrosirius red) and (h) fibroblasts (αSMA^+) in tumors from mice treated with a combination of vehicle or lorlatinib and PBS or gemcitabine. Five (Ly6G and aSMA) or eight (Ki67 and picrosirius red) images were acquired per mouse and used for quantifications (n = 8 mice per condition; data are from two independent experiments). Scale bars represent 100 μm (e, f, h) or 250 μm (g). Box-and-whisker plot shows the median (line), mean (plus sign), 25th and 75th percentiles (box) and 5th and 95th percentiles (whiskers); hypothesis testing performed using two-way ANOVA with Tukey's method for multiple comparisons.

Supplementary Table 1.

Antibodies for flow cytometry

Name	Host	Clone	Company	Cat. No.	Dilution
PE/Cy7 anti-mouse CD45	Rat	30-F11	Biolegend	103114	1:100 (FC)
PE anti-mouse/human CD11b	Rat	M1/70	Biolegend	101208	1:100 (FC)
FITC anti-mouse/human CD11b	Rat	M1/70	Biolegend	101206	1:100 (FC)
PE anti-mouse F4/80	Rat	BM8	Biolegend	123110	1:100 (FC)
APC anti-mouse F4/80	Rat	BM8	Biolegend	122116	1:100 (FC)
PerCP anti-mouse Ly-6C	Rat	HK1.4	Biolegend	128028	1:100 (FC)
APC anti-mouse Ly6G	Rat	1A8	Biolegend	127614	1:100 (FC)
PE anti-mouse CD3e	Armenian Hamster	145-2C11	Biolegend	100308	1:100 (FC)
PerCP anti-mouse CD4	Rat	RM4-5	Biolegend	100537	1:100 (FC)
APC anti-mouse CD8	Rat	53-6.7	Biolegend	100712	1:100 (FC)
APC anti-mouse/human CD45R/B220	Rat	RA3-6B2	Biolegend	103212	1:100 (FC)
PerCP anti-mouse Nk-1.1	Mouse	PK136	Biolegend	108726	1:100 (FC)
Brilliant Violet 421™ anti-mouse/human CD45R/B220	Rat	RA3-6B2	Biolegend	103239	1:100 (FC)
Brilliant Violet 421™ anti-mouse NK-1.1	Rat	PK136	Biolegend	108731	1:100 (FC)
Brilliant Violet 421™ anti-mouse CD90.2 (Thy-1.2)	Rat	53-2.1	Biolegend	140327	1:100 (FC)
Brilliant Violet 421™ anti-mouse F4/80	Rat	BM8	Biolegend	123131	1:100 (FC)
Brilliant Violet 421™ anti-mouse I-A/I-E	Rat	M5/114.15.2	Biolegend	107631	1:100 (FC)
PE anti-mouse CD117 (c-Kit)	Rat	2B8	Biolegend	105808	1:100 (FC)
PerCP/Cyanine5.5 anti-mouse CD115 (CSF-1R)	Rat	AFS98	Biolegend	135526	1:100 (FC)
BD Pharmingen™ PerCP-Cy™5.5 Rat Anti-Mouse Siglec-F	Rat	E50-2440	BD Pharmingen	565526	1:100 (FC)
APC/Cyanine7 anti-mouse Ly-6G/Ly-6C (Gr-1)	Rat	RB6-8C5	Biolegend	108424	1:100 (FC)

PE/Cy7 anti-mouse CD182 (CXCR2)	Rat	SA044G4	Biolegend	149316	1:100 (FC)
Brilliant Violet 711™ anti-mouse CD184 (CXCR4)	Rat	L276F12	Biolegend	146517	1:100 (FC)
PE/Cy7 anti-mouse CD16/32	Rat	S17011E	Biolegend	156609	1:100 (FC)
Alexa Fluor® 647 Rat anti-Mouse CD34	Rat	RAM34	BD Pharmingen	560233	1:100 (FC)
APC/Cyanine7 anti-mouse CD69	Hamster	H1.2F3	Biolegend	104525	1:100 (FC)
FITC anti-mouse/human CD44	Rat	IM7	Biolegend	103022	1:100 (FC)

Supplementary Table 2.

Antibodies for immunohistochemistry, immunofluorescence & western blotting

Name	Host	Clone	Company	Cat. No.	Dilution
F4/80	Rat	BM8	ThermoFisher Scientific	14-4801-85	1:100 (IF)
Ly6G	Rat	1A8	Biolegend	127602	1:50 (IF)
αSMA	Rabbit		Abcam	ab5694	1:200 (IF), 1:1000 (IHC)
CD8a	Rabbit	D4W2Z	Cell Signaling	98941	1:200 (IF)
Ki67	Rabbit	D3B5	Cell Signaling	12202	1:100 (IHC)
Cleaved Caspase 3 (Asp175)	Rabbit		Cell Signaling	9661	1:100 (IHC)
p-tyr705 STAT3	Rabbit	D3A7	Cell Signaling	9145	1:1000 (WB)
STAT3	Rabbit	C-20	Santa Cruz	sc-482	1:1000 (WB)
p-tyr694 STAT5	Rabbit	C71E5	Cell Signaling	9314	1:1000 (WB)
STAT5	Rabbit	D206Y	Cell Signaling	94205	1:1000 (WB)
Vinculin	Mouse	hVIN-1	Sigma	V9131	1:200 (WB)
Tubulin	Mouse	DM1A	Sigma	T6199	1:1000 (WB)
Anti-Rabbit IgG H&L (Alexa Fluor® 488)	Goat		Abcam	ab150077	1:500 (IF)
Anti-Rat IgG H&L (Alexa Fluor® 555)	Goat		Abcam	ab150158	1:500 (IF)
Anti-rabbit HRP	Goat		Dako	G-21234	1:1000 (WB)
Anti-mouse HRP	Rabbit		Dako	P0260	1:1000 (WB)
Dako EnVision+ System- HRP Labelled Polymer Anti-mouse			Dako	K4001	
Dako EnVision+ System- HRP Labelled Polymer Anti-Rabbit			Dako	K4003	

Supplementary References

1. Evrard, M. *et al.* Developmental Analysis of Bone Marrow Neutrophils Reveals Populations Specialized in Expansion, Trafficking, and Effector Functions. *Immunity* **48**, 364-379.e8 (2018).
2. Bagger, F. O., Kinalis, S. & Rapin, N. BloodSpot: A database of healthy and malignant haematopoiesis updated with purified and single cell mRNA sequencing profiles. *Nucleic Acids Res.* **47**, D881–D885 (2019).
