# Supplementary material for: Suppression of tumor-associated neutrophils by lorlatinib attenuates pancreatic cancer growth and improves treatment with immune checkpoint blockade
Source: Nat Commun. 2021 Jun 7;12:3414. doi: 10.1038/s41467-021-23731-7 (PMC8184753; doi:10.1038/s41467-021-23731-7)
Supplement: Supplementary file 2 — Descriptions of Additional Supplementary Files [file 41467_2021_23731_MOESM2_ESM.pdf]

Descriptions of Additional Supplementary Files

Supplementary Data 1
Description: Predicted kinase activity in murine neutrophils after stimulation with control or KPC-CM for 1 hour.

Supplementary Data 2
Description: Peptides used for prediction of kinase activity in murine neutrophils after stimulation with control or KPC-CM for 1 hour.

Supplementary Data 3
Description: Predicted kinase activity in murine neutrophils after stimulation with KPC-CM for 1 hour in the presence of vehicle or lorlatinib.

Supplementary Data 4
Description: Peptides used for prediction of kinase activity in murine neutrophils after stimulation with KPC-CM for 1 hour in the presence of vehicle or lorlatinib.

Supplementary Data 5
Description: Predicted kinase activity in murine neutrophils isolated from tumor-bearing KPC mice or tumor-free control mice.
