# Supplementary material for: Suppression of tumor-associated neutrophils by lorlatinib attenuates pancreatic cancer growth and improves treatment with immune checkpoint blockade
Source: Nat Commun. 2021 Jun 7;12:3414. doi: 10.1038/s41467-021-23731-7 (PMC8184753; doi:10.1038/s41467-021-23731-7)
Supplement: Supplementary file 8 — Reporting Summary [file 41467_2021_23731_MOESM8_ESM.pdf]

natureresearch

Corresponding author(s): Sebastian Rune Nielsen
Janine Terra Erler

Last updated by author(s): Apr 12, 2021

Reporting Summary

Nature Research wishes to improve the reproducibility of the work that we publish. This form provides structure for consistency and transparency in reporting. For further information on Nature Research policies, see Authors & Referees and the Editorial Policy Checklist .

Statistics

For all statistical analyses, confirm that the following items are present in the figure legend, table legend, main text, or Methods section.

n/a	Confirmed	
☐	☒	The exact sample size (*n*) for each experimental group/condition, given as a discrete number and unit of measurement
☐	☒	A statement on whether measurements were taken from distinct samples or whether the same sample was measured repeatedly
☐	☒	The statistical test(s) used AND whether they are one- or two-sided<br>*Only common tests should be described solely by name; describe more complex techniques in the Methods section.*
☒	☐	A description of all covariates tested
☐	☒	A description of any assumptions or corrections, such as tests of normality and adjustment for multiple comparisons
☐	☒	A full description of the statistical parameters including central tendency (e.g. means) or other basic estimates (e.g. regression coefficient) AND variation (e.g. standard deviation) or associated estimates of uncertainty (e.g. confidence intervals)
☐	☒	For null hypothesis testing, the test statistic (e.g. *F*, *t*, *r*) with confidence intervals, effect sizes, degrees of freedom and *P* value noted<br>*Give P values as exact values whenever suitable.*
☒	☐	For Bayesian analysis, information on the choice of priors and Markov chain Monte Carlo settings
☒	☐	For hierarchical and complex designs, identification of the appropriate level for tests and full reporting of outcomes
☒	☐	Estimates of effect sizes (e.g. Cohen's *d*, Pearson's *r*), indicating how they were calculated

Our web collection on statistics for biologists contains articles on many of the points above.

Software and code

Policy information about availability of computer code

Data collection	- Colonies in the colony formation assay and migrated cells in the transwell migration assay was imaged using brightfield microscope (Olympus CKX53 with Olympus DP22 camera) with Olympus cellSens Entry software version v.1.16.<br>- Immunofluorescence images were imaged using an SP8 confocal microscope (Leica), software Leica Application Suite X (LAS X) version 3.5.5.19976.<br>- H&E, picrosirius red and immunohistochemistry slides were imaged using a Hamamatsu Photonics slide scanner with NanoZoomer Digital Pathology (NDP) Scan software; NDP.scan version 2.5.90.<br>- Extinction values in the proliferation assays were acquired using a Spectra Max Paradigm plate reader from Molecular Devices, SoftMax Pro6.5.l software.<br>- Kinase activity was determined using PamChip-4 on a Pamstation®12 instrument from PamGene International BV with Upstream Kinase Analysis App (v.2018), BioNavigator6 (version 6.3.67.0) and Evolve2.2 (release 0.08) software.<br>- RT-qPCR data were acquired using a LightCycler 480 II (Roche) with LightCycler software (version 1.5.1)<br>- All flow cytometry data acquisition and cell sorting experiments were performed using a BD FACSAria III Cell Sorter (Model No: 648282–10–010010–X –X–X) with BD FACSDiva (v8.0.3) software.
Data analysis	- Quantification of H&E staining was performed using NDP.view2 Viewing software (Hamamatsu, U12388-01).<br>- Picrosirius red, immunohistochemistry and Immunofluorescence images were analyzed using ImageJ (Fiji Version 2.1.0/1.53c)<br>- Signal intensities of phosphorylated bait peptides were analyzed in BioNavigator6 software (version 6.3.67.0) and prediction of kinase activity was analyzed using Upstream Kinase Analysis App (PamGene, v.2018).<br>- Flow cytometry data were analyzed using BD FACSDiva (v8.0.3) software.<br>- qRT-PCR was analyzed using the LightCycler 480 software (version 1.5.1).<br>- All statistical analysis were performed using GraphPad Prism 9.0.2

For manuscripts utilizing custom algorithms or software that are central to the research but not yet described in published literature, software must be made available to editors/reviewers. We strongly encourage code deposition in a community repository (e.g. GitHub). See the Nature Research guidelines for submitting code & software for further information.

Data

Policy information about availability of data

All manuscripts must include a data availability statement. This statement should provide the following information, where applicable:

- Accession codes, unique identifiers, or web links for publicly available datasets
- A list of figures that have associated raw data
- A description of any restrictions on data availability

The transcriptome data used in this study are available in the GEO database under accession code: GSE109467 (bulk RNAseq from murine bone marrow neutrophil subsets) or BloodSpot database: http://servers.binf.ku.dk/bloodspot with HemaExplorer dataset (bulk RNAseq from human bone marrow subsets). Full list of the predicted kinase activity including the detected level of phosphorylation for each bait peptide is provided in supplementary tables 3-7. The remaining data are available within the Article, Supplementary Information, or available from the authors upon reasonable request.

Field-specific reporting

Please select the one below that is the best fit for your research. If you are not sure, read the appropriate sections before making your selection.

☒ Life sciences ☐ Behavioural & social sciences ☐ Ecological, evolutionary & environmental sciences

For a reference copy of the document with all sections, see nature.com/documents/nr-reporting-summary-flat.pdf

Life sciences study design

All studies must disclose on these points even when the disclosure is negative.

Sample size	We did not use any statistical methods to predetermine sample size. For ex vivo or in vitro experiments, we used 3-4 biological replicates as this sample size is sufficient for a statistical analysis. For experiments in vivo, the number of animals per treatment group was determined based on our prior studies using the same models (Nielsen et al., Nature Cell Biology 2016; Ireland et al., Cancer Research 2017). We estimated that 5 animals per group would account for the variability typically seen in tumor growth.
Data exclusions	No data were excluded from analysis.
Replication	Unless otherwise specified in the figure legends, experiments in vivo were reproduced in at least two independent experiments and all experiments in vitro were reproduced in at least three independent experiments.
Randomization	Mice were randomized before tumor inoculation and again before allocation to treatment groups. All cells in an experiment was prepared from the same source before seeding. After attachment, cells were allocated to different treatment groups in a random order.
Blinding	For the following, experiments were performed by one researcher and analyzed by a different researcher in a blinded manner: neutrophil transwell migration, cancer cell-neutrophil co-cultures, kinase activity, immunostainings and colony formation. All other experiments were unblinded because the analyses were performed using quantifiable parameters such that no bias was involved. For example, mice were randomly allocated to treatment groups, euthanised after same time and tumor weight was used for monitoring the size of orthotopic tumors; total weight of livers were normalized to the weight of each mouse and size of metastatic lesions were quantified to determine metastatic burden; time of death was recorded in the survival study; and the percentage of cells were derived from flow cytometry analysis using the same gating strategy for each sample.

Reporting for specific materials, systems and methods

We require information from authors about some types of materials, experimental systems and methods used in many studies. Here, indicate whether each material, system or method listed is relevant to your study. If you are not sure if a list item applies to your research, read the appropriate section before selecting a response.

Materials & experimental systems

n/a	Involved in the study	
☐	☒	Antibodies
☐	☒	Eukaryotic cell lines
☒	☐	Palaeontology
☐	☒	Animals and other organisms
☒	☐	Human research participants
☒	☐	Clinical data

Methods

n/a	Involved in the study	
☒	☐	ChIP-seq
☐	☒	Flow cytometry
☒	☐	MRI-based neuroimaging

Antibodies

Antibodies used

Antibodies used (name; company; catalog number; clone; dilution):
Anti-mouse Ly6G (BioXCell; Cat#BP0075-1; 1A8; 200ug/dose for depletion)
Anti-mouse PD-1 (BioXCell; Cat#BE0146; RMP1-14; 250ug/dose for neutralisation)
IgG2a isotype control (BioXCell; Cat#BP0089; 2A3; 200ug/dose or 250ug/dose in vivo)
PE/Cy7 anti-mouse CD45 (Biolegend; 103114; 30-F11; 1:100 for flow cytometry)
PE anti-mouse/human CD11b (Biolegend; 101208; M1/70; 1:100 for flow cytometry)
FITC anti-mouse/human CD11b (Biolegend, 101206; M1/70; 1:100 for flow cytometry)
PE anti-mouse F4/80 (Biolegend; 123110; BM8; 1:100 for flow cytometry)
APC anti-mouse F4/80 (Biolegend; 123116; BM8; 1:100 for flow cytometry)
PerCP anti-mouse Ly-6C (Biolegend; 128028; HK1.4; 1:100 for flow cytometry)
APC anti-mouse Ly6G (Biolegend; 127614; 1A8; 1:100 for flow cytometry)
PE anti-mouse CD3e (Biolegend; 100308; 145-2C11; 1:100 for flow cytometry)
PerCP anti-mouse CD4 (Biolegend; 100537; RM4-5; 1:100 for flow cytometry)
APC anti-mouse CD8 (Biolegend;100712; 53-6.7; 1:100 for flow cytometry)
APC anti-mouse/human CD45R/B220 (Biolegend; 103212; RA3-6B2; 1:100 for flow cytometry)
PerCP anti-mouse Nk-1.1 (Biolegend; 108726; PK136; 1:100 for flow cytometry)
Brilliant Violet 421™ anti-mouse/human CD45R/B220 (Biolegend; 103239; RA3-6B2; 1:100 for flow cytometry)
Brilliant Violet 421™ anti-mouse NK-1.1 (Biolegend; 108731; PK136; 1:100 for flow cytometry)
Brilliant Violet 421™ anti-mouse CD90.2 (Thy-1.2) (Biolegend; 140327; 53-2.1; 1:100 for flow cytometry)
Brilliant Violet 421™ anti-mouse F4/80 (Biolegend; 123131; BM8; 1:100 for flow cytometry)
Brilliant Violet 421™ anti-mouse I-A/I-E (Biolegend; 107631; M5/114.15.2; 1:100 for flow cytometry)
PE anti-mouse CD117 (c-Kit) (Biolegend; 105808; 2B8; 1:100 for flow cytometry)
PerCP/Cyanine5.5 anti-mouse CD115 (CSF-1R) (Biolegend; 135526; AFS98; 1:100 for flow cytometry)
PerCP-Cy™5.5 anti-mouse Siglec-F (BD Pharmingen; 565526; E50-2440; 1:100 for flow cytometry)
APC/Cyanine7 anti-mouse Ly-6G/Ly-6C (Gr-1) (Biolegend; 108424; RB6-8C5; 1:100 for flow cytometry)
PE/Cy7 anti-mouse CD182 (CXCR2) (Biolegend; 149316; SA044G4; 1:100 for flow cytometry)
Brilliant Violet 711™ anti-mouse CD184 (CXCR4) (Biolegend; 146517; L276F12; 1:100 for flow cytometry)
PE/Cy7 anti-mouse CD16/32 (Biolegend; 156609; S17011E; 1:100 for flow cytometry)
Alexa Fluor® 647 Rat anti-Mouse CD34 (BD Pharmingen; 560233; RAM34; 1:100 for flow cytometry)
APC/Cyanine7 anti-mouse CD69 (Biolegend; 104525, H1.2F3; 1:100 for flow cytometry)
FITC anti-mouse/human CD44 (Biolegend; 103022; 1:100 for flow cytometry)
Anti-F4/80 (ThermoFisher Scientific; 14-4801-85; BM8; 1:100 for immunofluorescence)
Anti- Ly6G (Biolegend; 127602; 1A8; 1:50 for immunofluorescence)
Anti-aSMA (Abcam; ab5694; 1:200 for immunofluorescence)
Anti-CD8a (Cell Signaling; 98941; D4W2Z; 1:100 for immunofluorescence)
Anti-Ki67 (Cell Signaling; 12202S; D3B5; 1:100 for immunohistochemistry)
Anti-Cleaved Caspase 3 (Asp175) (Cell Signaling; 9661S; 1:100 for immunohistochemistry)
Anti-p-tyr705 STAT3(Cell Signaling; 9145S; D3A7; 1:1000 for western blotting)
Anti-STAT3 (Santa Cruz; sc-482; C-20; 1:1000 for western blotting)
Anti-p-tyr694 STAT5 (Cell Signaling; 9314S; C71E5; 1:1000 for western blotting)
Anti-STAT5 (Cell Signaling; 94205; D206Y; 1:1000 for western blotting)
Anti-Vinculin (Sigma-Aldrich; V9131; hVIN-1; 1:200 for western blotting)
Anti-Tubulin (Sigma-Aldrich; T6199; DM1A; 1:1000 for western blotting)
Goat Anti-Rabbit IgG H&L (Alexa Fluor® 488) (Abcam; ab150077; 1:500 for immunofluorescence)
Goat Anti-Rat IgG H&L (Alexa Fluor® 555) (Abcam; ab150158; 1:500 for immunofluorescence)
Dako EnVision+ System- HRP Labelled Polymer Anti-mouse (Dako; K4001; immunohistochemistry)
Dako EnVision+ System- HRP Labelled Polymer Anti-Rabbit (Dako; K4003; immunohistochemistry)
Goat Anti-rabbit HRP (Dako; G-21234; 1:1000 for WB)
Rabbit Anti-mouse HRP (Dako; P0260; 1:1000 for WB)

Validation

Antibody validation information can be found on manufacturers' website:
- Anti-mouse Ly6G: https://bxcell.com/product/invivoplus-anti-m-ly-6g-2/
- Anti-mouse PD-1: https://bxcell.com/product/invivomab-anti-m-pd-1/
- IgG2a isotype control: https://bxcell.com/product/invivoplus-rat-igg2a-isotype-control-anti-trinitrophenol/
- PE/Cy7 anti-mouse CD45: https://www.biolegend.com/en-us/products/pe-cyanine7-anti-mouse-cd45-antibody-1903?GroupID=GROUP20
- PE anti-mouse/human CD11b: https://www.biolegend.com/en-us/products/pe-anti-mouse-human-cd11b-antibody-349
- FITC anti-mouse/human CD11b: https://www.biolegend.com/en-us/products/fitc-anti-mouse-human-cd11b-antibody-347
- PE anti-mouse F4/80: https://www.biolegend.com/en-us/products/pe-anti-mouse-f4-80-antibody-4068
- APC anti-mouse F4/80: https://www.biolegend.com/en-us/products/apc-anti-mouse-f4-80-antibody-4071
- PerCP anti-mouse Ly-6C: https://www.biolegend.com/en-us/products/percp-anti-mouse-ly-6c-antibody-7166
- APC anti-mouse Ly6G: https://www.biolegend.com/en-us/products/apc-anti-mouse-ly-6g-antibody-6115

- PE anti-mouse CD3e: https://www.biolegend.com/en-us/products/pe-anti-mouse-cd3epsilon-antibody-25
- PerCP anti-mouse CD4: https://www.biolegend.com/en-us/products/percp-anti-mouse-cd4-antibody-4229
- APC anti-mouse CD8: https://www.biolegend.com/en-us/products/apc-anti-mouse-cd8a-antibody-150
- APC anti-mouse/human CD45R/B220: https://www.biolegend.com/en-us/products/apc-anti-mouse-human-cd45r-b220-antibody-442
- PerCP anti-mouse Nk-1.1: https://www.biolegend.com/en-us/products/percp-anti-mouse-nk-1-1-antibody-4288
- Brilliant Violet 421™ anti-mouse/human CD45R/B220: https://www.biolegend.com/en-us/products/brilliant-violet-421-anti-mouse-human-cd45r-b220-antibody-7158
- Brilliant Violet 421™ anti-mouse NK-1.1: https://www.biolegend.com/en-us/products/brilliant-violet-421-anti-mouse-nk-1-1-antibody-7150
- Brilliant Violet 421™ anti-mouse CD90.2 (Thy-1.2): https://www.biolegend.com/en-us/products/brilliant-violet-421-anti-mouse-cd90-2-thy-1-2-antibody-14309
- Brilliant Violet 421™ anti-mouse F4/80: https://www.biolegend.com/en-us/products/brilliant-violet-421-anti-mouse-f4-80-antibody-7199
- Brilliant Violet 421™ anti-mouse I-A/I-E: https://www.biolegend.com/en-us/products/brilliant-violet-421-anti-mouse-i-a-i-e-antibody-7147
- PE anti-mouse CD117 (c-Kit): https://www.biolegend.com/en-us/products/pe-anti-mouse-cd117-c-kit-antibody-75
- PerCP/Cyanine5.5 anti-mouse CD115 (CSF-1R):https://www.biolegend.com/en-us/products/percp-cyanine5-5-anti-mouse-cd115-csf-1r-antibody-12377
- PerCP-Cy™5.5 anti-mouse Siglec-F: https://www.bdbiosciences.com/eu/reagents/research/antibodies-buffers/immunology-reagents/anti-mouse-antibodies/cell-surface-antigens/percp-cy55-rat-anti-mouse-siglec-f-e50-2440/p/565526
- APC/Cyanine7 anti-mouse Ly-6G/Ly-6C (Gr-1): https://www.biolegend.com/en-us/products/apc-cyanine7-anti-mouse-ly-6gly-6c-gr-1-antibody-3935
- PE/Cy7 anti-mouse CD182 (CXCR2): https://www.biolegend.com/en-us/products/pe-cyanine7-anti-mouse-cd182-cxcr2-antibody-16122
- Brilliant Violet 711™ anti-mouse CD184 (CXCR4): https://www.biolegend.com/en-us/products/brilliant-violet-711-anti-mouse-cd184-cxcr4-antibody-14645
- PE/Cy7 anti-mouse CD16/32: https://www.biolegend.com/en-us/products/pe-cyanine7-anti-mouse-cd1632-antibody-17483
- Alexa Fluor® 647 Rat anti-Mouse CD34: https://www.bdbiosciences.com/eu/applications/research/stem-cell-research/cancer-research/mouse/alexa-fluor-647-rat-anti-mouse-cd34-ram34/p/560233
- APC/Cyanine7 anti-mouse CD69: https://www.biolegend.com/en-us/products/apc-cyanine7-anti-mouse-cd69-antibody-6986
- FITC anti-mouse/human CD44: https://www.biolegend.com/en-us/products/fitc-anti-mouse-human-cd44-antibody-314
- Anti-F4/80: https://www.thermofisher.com/antibody/product/F4-80-Antibody-clone-BM8-Monoclonal/14-4801-82
- Anti- Ly6G: https://www.biolegend.com/en-us/products/purified-anti-mouse-ly-6g-antibody-4767
- Anti-aSMA: https://www.abcam.com/alpha-smooth-muscle-actin-antibody-ab5694.html
- Anti-Ki67: https://www.cellsignal.com/products/primary-antibodies/ki-67-d3b5-rabbit-mab-mouse-preferred-ihc-formulated/12202
- Anti-Cleaved Caspase 3 (Asp175): https://www.cellsignal.com/products/primary-antibodies/cleaved-caspase-3-asp175-antibody/9661
- Anti-p-tyr705 STAT3: https://www.cellsignal.com/products/primary-antibodies/phospho-stat3-tyr705-d3a7-xp-rabbit-mab/9145
- Anti-STAT3: https://www.scbt.com/p/stat3-antibody-c-20
- Anti-p-tyr694 STAT5: https://www.cellsignal.com/products/primary-antibodies/phospho-stat5-tyr694-c71e5-rabbit-mab/9314?site-search-type=Products&N=4294956287&Ntt=9314s&fromPage=plp&_requestid=980645
- Anti-STAT5: https://www.cellsignal.com/products/primary-antibodies/stat5-d2o6y-rabbit-mab/94205
- Anti-Vinculin: https://www.sigmaaldrich.com/catalog/product/sigma/v9131?lang=en&region=US
- Anti-Tubulin: https://www.sigmaaldrich.com/catalog/product/sigma/t6199?lang=en&region=US&cm_sp=Insite-_-caSrpResults_srpRecs_srpModel_t6199-_-srpRecs3-1
- Goat Anti-Rabbit IgG H&L (Alexa Fluor® 488): https://www.abcam.com/goat-rabbit-igg-hl-alexa-fluor-488-ab150077.html
- Goat Anti-Rat IgG H&L (Alexa Fluor® 555): https://www.abcam.com/goat-rat-igg-hl-alexa-fluor-555-ab150158.html

Eukaryotic cell lines

Policy information about cell lines

Cell line source(s)	KPC mT4 cells and cancer-associated fibroblast cell lines 17964-56 and 19238-43 were generated from pancreatic tumors from KrasG12D/+;Trp53R172H/+;Pdx1-Cre (KPC) mice in the Tuveson Lab (Boj et al., Cell 2014; Öhlund et al., The Journal of Experimental Medicine 2017). Colorectal cancer cells were established from colorectal tumors in villinCreER Apcfl/fl KrasG12D/+ Trp53fl/fl Trgfbrlfl/fl mice in the Sansom Lab (Jackstadt et al., Cancer Cell 2019).
Authentication	Authentication of KPC mT4 cell line was performed by confirmation of KrasG12D recombination and loss of heterozygosity of the wild-type Trp53 allele by PCR (see Boj et al., Cell 2014). Cancer-associated fibroblasts were confirmed to be wild-type for Kras by PCR and express fibroblast markers (see Öhlund et al., The Journal of Experimental Medicine 2017). Colorectal cancer cells were established from tumors in mice with the genotype listed above and maintained in the Sansom lab.
Mycoplasma contamination	All cell lines were routinely tested for mycoplasma and murine pathogen contamination. No contamination was found at any time point during the course of the study.

Commonly misidentified lines
(See ICLAC register)

No commonly misidentified cell lines were used.

Animals and other organisms

Policy information about studies involving animals; ARRIVE guidelines recommended for reporting animal research

Laboratory animals

Mice used for orthotopic and intrasplenic injections of KPC mT4 cells were females of C57BL/6 background. Mice used for intrasplenic injections of colorectal cancer cells were males on a C57BL/6 background. All mice were between 6-12 weeks old at the start of an experiment.
KrasLSL-G12D/+;Trp53LSL-R172H/+;Pdx1-Cre mice were bred in Copenhagen on a C57BL/6 background or Glasgow on a mixed background. KPC mice were monitored at least 3 times weekly and culled when exhibiting symptoms of PDAC. Both male and female KPC mice were included in the study.
All animals were housed in University of Copenhagen or University of Glasgow SPF animal facilities, in temperatures 20-22 celsius, humidity 30-70% and 12-hour light/12-hour dark cycles.

Wild animals

Study did not involve wild animals.

Field-collected samples

Study did not involve field-collected samples.

Ethics oversight

All experiments were carried out under authorization and guidance from the Danish Inspectorate for Animal Experimentation (License 2017-15-0201-01265) or UK Home Office license UK Home Office license (Project License 70/8646) and approved by the University of Glasgow Animal Welfare and Ethical Review Board.

Note that full information on the approval of the study protocol must also be provided in the manuscript.

Flow Cytometry

Plots

Confirm that:

- [x] The axis labels state the marker and fluorochrome used (e.g. CD4-FITC).
- [x] The axis scales are clearly visible. Include numbers along axes only for bottom left plot of group (a 'group' is an analysis of identical markers).
- [x] All plots are contour plots with outliers or pseudocolor plots.
- [x] A numerical value for number of cells or percentage (with statistics) is provided.

Methodology

Sample preparation

Flow cytometry analysis was performed on blood, bone marrow, primary tumors and metastatic livers. Blood samples were collected by cardiac puncture, subjected to erythrolysis and stained according to standard protocols. Bone marrow was collected by flushing the femur and tibia, subjected to erythrolysis and stained according to standard protocols. Liver and tumor samples were digested into a single cell suspension with collagenase mix, washed and stained accordingly.

Instrument

Flow cytometry analysis and cell sorting were performed on BD FACS Aria III.

Software

BD FACSDiva (v8.0.3) software was used to analyze all cytometry data

Cell population abundance

Neutrophils were isolated from murine bone marrow by density gradient centrifugation and purified by negative magnetic bead isolation (to deplete non-neutrophils). Purity of isolated cells were determined by flow cytometry and is shown in supplement figure 1A. Purity of cell sorting was performed by analyzing samples from the sorted cell fractions and was determined to be above 90%

Gating strategy

FSC/SSC gates were chosen to eliminate debris and doublets. Dead cells were eliminated by excluding SYTOX+ cells.
Within a single experiment, gating was determined based on sample gating of a random sample, before gates were applied to all plots for final analysis. Examples of gating strategy are presented in the Supplementary Fig. 3, and 8.

- [x] Tick this box to confirm that a figure exemplifying the gating strategy is provided in the Supplementary Information.
